# Structural Characterization of the Reaction and Substrate Specificity Mechanisms of Pathogenic Fungal Acetyl-CoA Synthetases

**DOI:** 10.1021/acschembio.1c00484

**Published:** 2021-08-09

**Authors:** Andrew J. Jezewski, Katy M. Alden, Taiwo E. Esan, Nicholas D. DeBouver, Jan Abendroth, Jameson C. Bullen, Brandy M. Calhoun, Kristy T. Potts, Daniel M. Murante, Timothy J. Hagen, David Fox, Damian J. Krysan

**Affiliations:** † Department of Pediatrics Carver College of Medicine, University of Iowa, Iowa City, Iowa 52242, United States; ‡ Department of Chemistry and Biochemistry, Northern Illinois University, DeKalb, Illinois 60115, United States; § UCB Pharma, 7869 NE Day Road West, Bainbridge Island, Washington 98110, United States; ∥ Beryllium Discovery Corp., 7869 NE Day Road West, Bainbridge Island, Washington 98110, United States; ⊥ Seattle Structural Genomics Center for Infectious Disease (SSGCID), Seattle, Washington 98109, United States; # Microbiology/Immunology, Carver College of Medicine, University of Iowa, Iowa City, Iowa 52242, United States

## Abstract

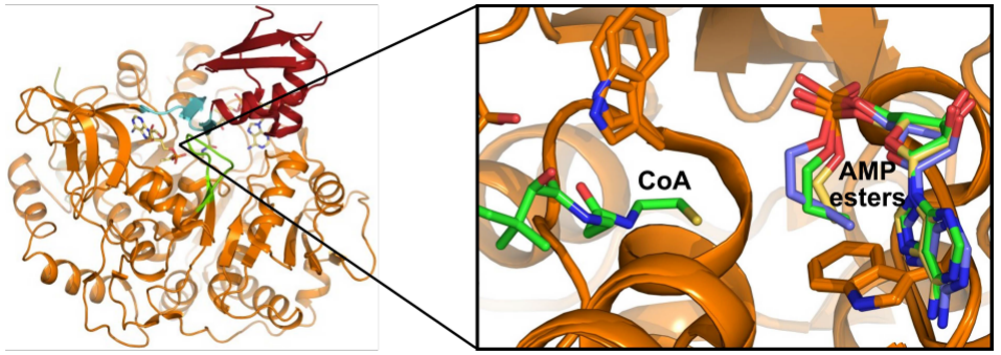

Acetyl CoA synthetases (ACSs) are **A**cyl-CoA/**N**RPS/**L**uciferase (ANL) superfamily enzymes that couple acetate with CoA to generate acetyl CoA, a key component of central carbon metabolism in eukaryotes and prokaryotes. Normal mammalian cells are not dependent on ACSs, while tumor cells, fungi, and parasites rely on acetate as a precursor for acetyl CoA. Consequently, ACSs have emerged as a potential drug target. As part of a program to develop antifungal ACS inhibitors, we characterized fungal ACSs from five diverse human fungal pathogens using biochemical and structural studies. ACSs catalyze a two-step reaction involving adenylation of acetate followed by thioesterification with CoA. Our structural studies captured each step of these two half-reactions including the acetyl-adenylate intermediate of the first half-reaction in both the adenylation conformation and the thioesterification conformation and thus provide a detailed picture of the reaction mechanism. We also used a systematic series of increasingly larger alkyl adenosine esters as chemical probes to characterize the structural basis of the exquisite ACS specificity for acetate over larger carboxylic acid substrates. Consistent with previous biochemical and genetic data for other enzymes, structures of fungal ACSs with these probes bound show that a key tryptophan residue limits the size of the alkyl binding site and forces larger alkyl chains to adopt high energy conformers, disfavoring their efficient binding. Together, our analysis provides highly detailed structural models for both the reaction mechanism and substrate specificity that should be useful in designing selective inhibitors of eukaryotic ACSs as potential anticancer, antifungal, and antiparasitic drugs.

## Introduction

Acetyl CoA is a key molecule in biology that plays roles in cellular energetics, regulation of gene expression, post-translational modification of proteins, and lipid biosynthesis among other fundamental cellular functions.^1^ Eukaryotic cells generate acetyl CoA through multiple pathways including^2^ (1) pyruvate dehydrogenase (PDH) mediated conversion of pyruvate, (2) ATP-citrate lyase conversion of citrate, (3) β-oxidation of fatty acids, and (4) direct synthesis from acetate using acetyl CoA synthetases (ACSs). The relative contributions of these pathways vary with organism, environment, nutrient status, tissue type, and specific intracellular compartment.^2^ In humans, the predominant pathway appears to be ATP-citrate lyase in normal cells under nonstress conditions.^3^ Interestingly, recent studies have indicated that tumor cells are much more dependent on the acetyl CoA synthetase (ACSS2)-mediated conversion of acetate to acetyl CoA than nontransformed cells.^4,5^ In addition, ACSS2 has also been shown to promote the storage of fat and affects the distribution and utilization of lipids.^6^ These and other studies indicating a role for ACSS2 in histone modification and aging have prompted significant interest in the direct conversion of acetate to acetyl CoA by ACSs.

One of the reasons for interest in ACS is that there is no apparent phenotype in adult mice lacking ACSS2, indicating that this enzyme is not likely to be essential and, thus, raising its potential as a therapeutic target.^4−7^ Further supporting this concept is the fact that *acss2*^–/–^ mice have a reduced tumor burden in mouse models of cancer.^4,5^ Similarly, Huang et al. have suggested that ACSS2 may also be a target for the treatment of fatty liver disease based on its role in the regulation of fat storage.^8^ Our interest in this pathway is also related to the nonessentiality of the pathway in mammals. Specifically, ACS enzymes are essential in *Candida* spp., the most common human fungal pathogen.^9,10^ In addition, previous studies have indicated that they are required for virulence in *Cryptococcus neoformans*,^11^ one of the most important causes of lethal opportunistic infections in people living with HIV/AIDS. Recently, Ries et al. have shown that acetate utilization is also required for virulence in *Aspergillus fumigatus*, indicating that important mold pathogens may also be susceptible to inhibition of acetate-related enzymes.^12^ The potential of ACS as an antifungal drug target is supported by previous work from our laboratory showing that the activity of the molecule AR-12 against clinically relevant yeasts and molds was due, at least in part, to inhibition of fungal ACS.^13,14^ Taken together, these observations support the targeting of ACS as a therapeutic approach to a variety of human diseases.

**Figure 1:**
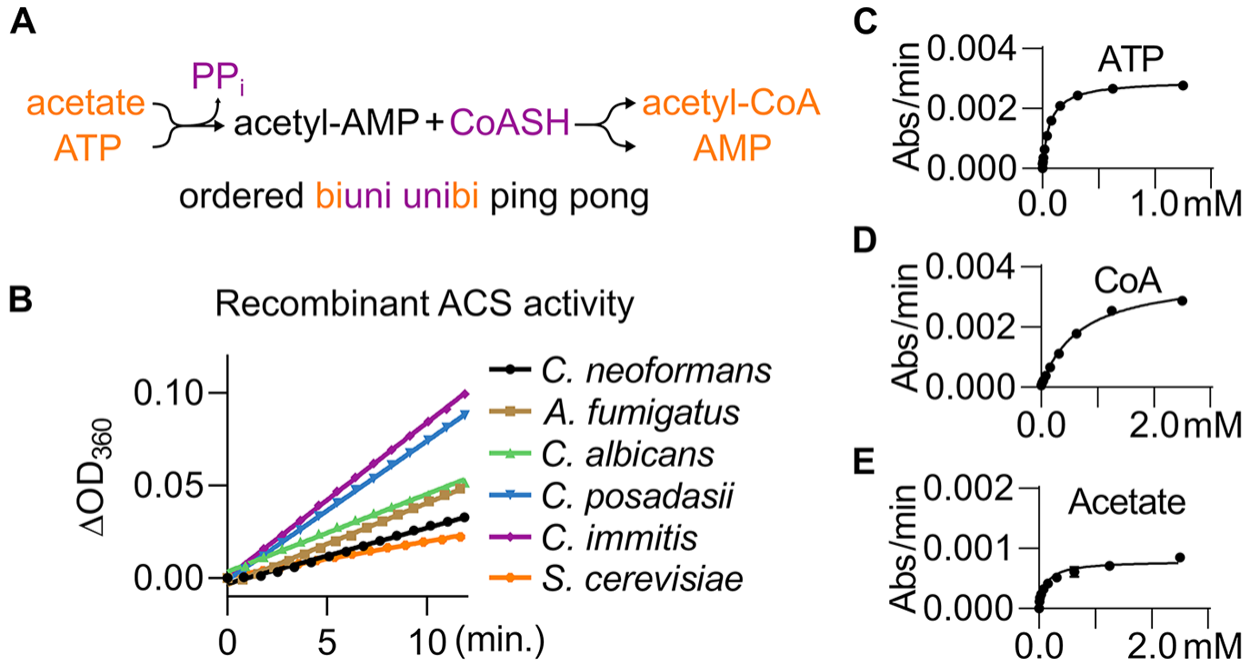
Expression and biochemical characterization of Acs1. (A) Reaction scheme of Acs1. (B) Progress curves for detecting the activity of purified recombinant Acs1 for six fungal enzymes using the EnzChek pyrophosphate detection kit (Thermo-Fisher). (C, D) Representative Michaelis–Menten curves for measuring substrate Km’s for *Cn*Acs1; values for all enzymes reported in Table 1.

To facilitate the systematic development of antifungal ACS inhibitors, we undertook the biochemical and structural characterization of ACS enzymes from phylogenetically disparate human fungal pathogens. ACS catalyzes a two-step reaction in which acetate is first condensed with ATP to generate the corresponding adenylated ester (Ac-AMP) along with the liberation of pyrophosphate.^15^ This highly reactive Ac-AMP intermediate then reacts with the thiol of coenzyme A to yield acetyl CoA and AMP (Figure 1A). Accordingly, ACSs are classified as adenylating enzymes and are part of the **A**cyl-CoA/**N**RPS/**L**uciferase (ANL) superfamily of enzymes.^16^ The *Saccharomyces cerevisiae* ACS, Acs1, was the founding member of this family, and its initial characterization by Berg et al. laid the foundation for subsequent mechanistic studies.^17^ The reaction proceeds in a biuni–unibi ping pong mechanism based on kinetic studies.^18^ A defining structural characteristic of the ANL-family enzymes is a 140° rotation of the C-terminal domain that results in opposing faces interacting with the active site depending upon which half-step of the reaction is occurring.^16^ The active site for the first adenylation step contains a critical lysine, which is acetylated by acetyl transferases, inhibiting the enzyme, while sirtuin deacetylases remove the acetyl group to activate the enzyme.^15^ As presented below, we describe X-ray crystal structures corresponding to each key intermediate in the ACS reaction, including the active site acetyl-lysine form. To our knowledge, these structures include the first example of an ACS with the acetyl-AMP intermediate in both conformations as well as the first apoenzyme structure for an ACS.

An important feature of an enzyme that is proposed as a drug target is the mechanism of its substrate specificity since the understanding of the determinants of specificity can guide the design of inhibitors. ACSs are part of a subfamily of ANL-enzymes that convert alkyl carboxylic acids to their corresponding acyl-CoA derivatives.^16^ ACSs have exquisite specificity for the acetate relative to alkyl carboxylates with even one more carbon atom.^19^ Previous genetic and structural studies of ACSs have indicated that an active site Trp limits the acetate binding pocket and prevents the efficient reaction of longer chain carboxylic acids. Specifically, as reported by Ingram-Smith et al.,^19^ mutation of the Trp residue to the much smaller Gly in *Methanothermobacter thermautotrophicus* ACS leads to a dramatic expansion in substrate specificity of ACS to larger alkyl carboxylic acids. Here, we used a set of methyl, ethyl, propyl, and butyl-AMP ester bisubstrate inhibitors to chemically probe the consequences of increasing the alkyl chain length on the structure of the active site. The resulting crystal structures clearly establish the role of an active site Trp as a “wall” that provides ACSs with a remarkable selectivity for acetate. Since eukaryotic ACS enzymes are well conserved, the highly detailed mechanistic information provided by these structural and biochemical studies should inform the analysis of other ACSs and facilitate their targeting with specific inhibitors.

## Results and Discussion

### Biochemical Characterization of Acetyl CoA Synthetases from the Human Fungal Pathogens *C. neoformans*, *C. albicans*, *A. fumigatus*, *C. immitis*, and *C. posadii*

The *S. cerevisiae* Acs1 was among the first ACSs to be characterized biochemically^17^ and structurally.^20^ To our knowledge, however, no ACSs from human fungal pathogens have been examined. We selected five ACS enzymes from three classes of human fungal pathogens: yeast (*C. neoformans* and *Candida albicans*), molds (*Aspergillus fumigatus*), and endemic fungi (*Coccidioides immitis* and *Coccidioides posadii*). The enzymes were expressed as N-terminal His8 fusion proteins in *E. coli* and purified by sequential immobilized metal affinity chromatography (IMAC) and size exclusion chromatography (SEC; Figure S1). To characterize the biochemical activity of the ACSs, we employed a coupled, continuous assay of pyrophosphate generation based on that reported by Comerford et al.^4^ for human acetyl CoA synthetase (ACSS2) and by Aldrich and Wilson for other adenylating enzymes.^21^ In this assay, pyrophosphate is first hydrolyzed to phosphate by pyrophosphatase, which is then coupled to MESG by phosphorylase. In the presence of ACS, production of a signal is dependent on the presence of all three substrates: ATP, acetate, and CoASH (Figure S2). As shown in Figure 1B, all five fungal pathogen ACS enzymes and *S. cerevisiae* Acs1 gave linear reaction progress curves over time. Using this assay, we characterized the substrate *K*_M_ and *V*_max_ values for each of the enzymes and, as a control, commercially available *S. cerevisiae* Acs1 (Figure 1C–E, Table 1). The *C. neoformans* enzyme showed the lowest specific activity of the five enzymes. In addition, the *K*_M_ values of the *Cn*Acs1 for both acetate and CoASH were higher than those for the other four enzymes; the *K*_M_ for ATP was similar for all five enzymes.

**Table 1:** Biochemical Properties of Recombinant Fungal Acs1^a^

species (Acs1)	specific activity (nmol min^–1^ mg^–1^)	ATP Km (μM)	CoA Km (μM)	acetate Km (μM)
*C. neoformans*	12	59 ± 10	880 ± 170	130 ± 40
*A. fumigatus*	70	42 ± 11	340 ± 26	18 ± 2
*C. albicans*	143	83 ± 14	471 ± 45	79 ± 7
*C. immitis*	102	102 ± 8	441 ± 45	32 ± 5
*C. posadasii*	292	117 ± 15	314 ± 32	27 ± 2
*S. cerevisiae*	836	161 ± 73	348 ± 43	63 ± 13

a All reported values are of three independent experimental replicates of technical duplicates, ±S.E.M.

### Overall Structures of Fungal ACS Enzymes Bound to Propyl-AMP

ACSs from two organisms, *S. cerevisiae*(20) and *Salmonella enterica*,^22^ have been previously crystallized and characterized structurally. With the goal of obtaining additional information into the mechanisms of the ACS reaction and substrate specificity, we sought to crystallize ACS enzymes from the five pathogenic fungal species. Because the N-terminal sequence prior to the start of the N-terminal ATP binding domain is poorly conserved across species, we designed N-terminally truncated Acs1 constructs to complement the full-length constructs used to biochemically characterize the enzymes. The truncated proteins were expressed and purified both to homogeneity as described above; the biochemical activity of the *Cn*Acs1 was similar to full-length enzymes. The two previous ACS structures contained either AMP^20^ or the substrate-based inhibitor propyl-**a**denosine **m**ono**p**hosphate ester (propyl-AMP; 22); no crystals of unliganded protein were obtained. We, therefore, performed initial crystallization experiments in the presence of propyl-AMP, which facilitated the crystallization of the *S. enterica* ACS. Crystals of full-length Acs1 were produced for all five species with the highest resolution structures obtained for *C. immitis* (1.80 Å), *C. neoformans* (1.95 Å), and *C. posadasii* (2.15 Å). Clear electron density for the propyl-AMP ligand as well as the coordinating protein residues and solvent were observed (Supplementary Table 1, Figure S8). *A. fumigatus* and *C. albicans* Acs1 were also solved but at a lower resolutions of 2.8 and 2.9 Å, respectively; however, we were able to confidently build the domains based on the high structural homology and the presence of the ligand.

**Figure 2:**
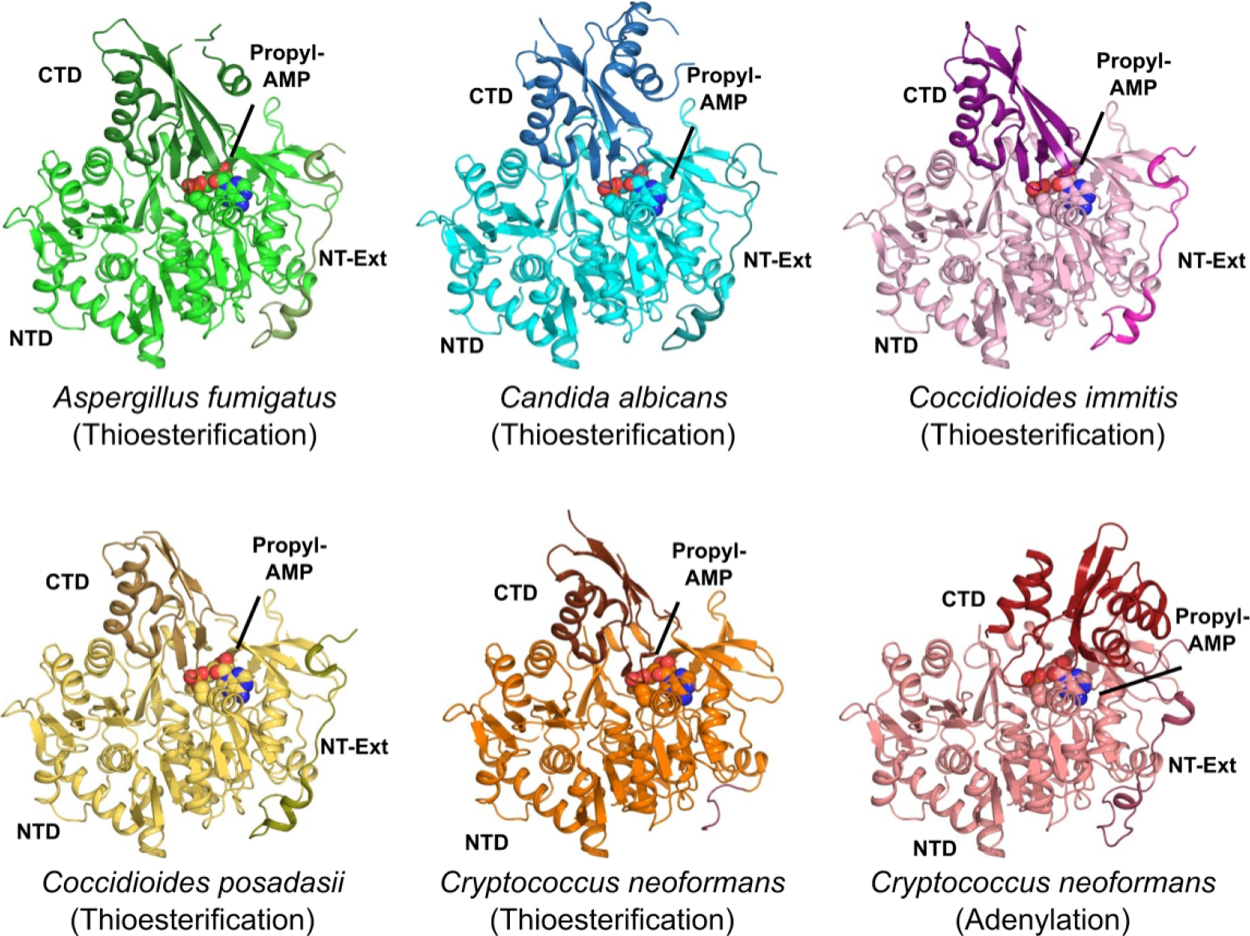
Overview of fungal orthologs of Acs1 bound to propyl-AMP. Structures shown such that the N-terminal domain (NTD) is light shades, while the C-terminal domain (CTD) and the N-terminal extension (NT-Ext) are in darker shades and the compounds are in spheres. *Aspergillus fumigatus* (green, PDB 7KDN, 2.8 Å), *Candida albicans* (blue, PDB 7KDS, 2.9 Å), *Coccidioides immitis* (pink, PDB 7KQ6, 1.8 Å), *Coccidioides posadasii* (yellow, PDB 7KCP, 2.15 Å), and *Cryptococcus neoformans* in two conformations—thioesterification (orange), adenylation (red, PDB 5IFI, 1.95 Å).

As discussed above,^16^ ANL-family enzymes undergo a large conformational change between the adenylation reaction (AD-conf) and the thioesterification reaction (TE-conf) during the two-step reaction sequence. The *Coccidioides* spp., *A. fumigatus* Acs1, and *C. albicans* Acs2 structures show the C-terminal domain in the TE-conf with one to six copies found in the asymmetric unit, typically arranged in a crystallographic trimer configuration, similar to *S. cerevisiae* (Figure 2).^21^ In contrast, *Cryptococcus* Acs1 preferred to crystallize with three copies in the asymmetric unit: two copies in the AD-conf and the third copy in the TE-conf. This crystal form allowed us to characterize bound substrates and inhibitors in both functionally relevant conformations within the same data set, an opportunity not observed in any other ACS or related structure to our knowledge. Our ability to isolate these two conformations suggests that formation of the acetyl-AMP intermediate in the adenylation conformation may represent a local minimum in the reaction sequence that is followed by the rotation of the CTD into the conformation that then facilitates the second, thioesterification of the acetyl-AMP. The observation of these two conformations also provides structural confirmation of previous mechanistic proposals that establishment of the TE-conf occurs before interaction of CoASH with the enzyme.

The structures determined include the canonical large N-terminal ATP-binding domain (NTD, 42–541, *Cryptococcus* numbering) and small C-terminal domain (CTD, 542–C-terminus). The CTD caps the ATP-binding pocket and presents the active site conserved lysine (Lys640) in the AD-conf and rotates ∼140° about a flexible hinge (Gly541) to form the CoA binding pocket. Generally, for all structures, electron density for the CTD had the highest B-factors, indicative of greater mobility. All fungal ACS enzymes included a poorly conserved N-terminal extension (NT-Ext) in the crystallization constructs. We found this extension to be ordered in most structures, traversing the outside of the NTD before it contacts the CTD in the AD-conf (Figures 2, S3). When the CTD rotates to the TE-conf, the N-terminal sequence loses contact and becomes partially disordered. While no regulatory function has been attributed to this NT-Ext, its ability to bind at the interface of the N- and C-terminal domains in the first reaction pose suggests that it may stabilize the AD-conf.

Despite relatively modest sequence identity across the five fungal ACSs, the overall structural homology between enzymes was remarkably high (>46% identity, 0.3–0.7 Å RMSD, Table S2).^22−24^ Within the ATP and acetate binding sites, all residues are conserved with the exception of Met440 in *Cryptococcus*, which is a glutamine in the other species (Table S3). A brief description of the propyl-AMP ester binding interactions to *Cryptococcus* Acs1 will be representative for the fungal species under investigation given that it was solved in the two active conformations. In brief, the adenine ring of the AMP propyl-ester inhibitor is bound on one side by the β18−α15 loop (Val411, Gly412, Glu413, and Pro414) and on the other side by the β19−α16 loop (Asp436, Thr437, and Tyr438), forming two hydrogen bonds with Asp436 and Thr437. The ribose ring 2′ and 3′ OH’s are hydrogen bonded to Asp527 and Arg542 (part of the hinge to the CTD) and reside near Met440 in *Cryptococcus*. The other four fungal species ACSs contain a conserved glutamine residue and form an additional hydrogen bond with the 3′ OH. The phosphate ester of the propyl-AMP inhibitor forms three hydrogen bonds to surrounding residues in the adenylating conformation, Thr441 (2×) and Trp334. The Trp334 indole NH is hydrogen bonded primarily to the phosphate oxygen in the AD-conf, but this interaction is lost in the TE-conf because it swings away from the propyl-AMP substrate-like inhibitor. In the TE-conf, the rotation of the CTD brings Arg553 into the active site, which forms two salt bridges with Glu442 and a hydrogen bond to the AMP phosphate, with a subsequent loss of the bond to Trp334. The aliphatic portion of the propyl-AMP ester pushes up against Trp439, acting as a substrate chain-length cap, and is surrounded by hydrophobic side chains from the “WIT” motif, 334–336, at the start of α11, and Val411.

### Structural Snapshots of the *C. neoformans* Acs1 Reaction Series

**Figure 3:**
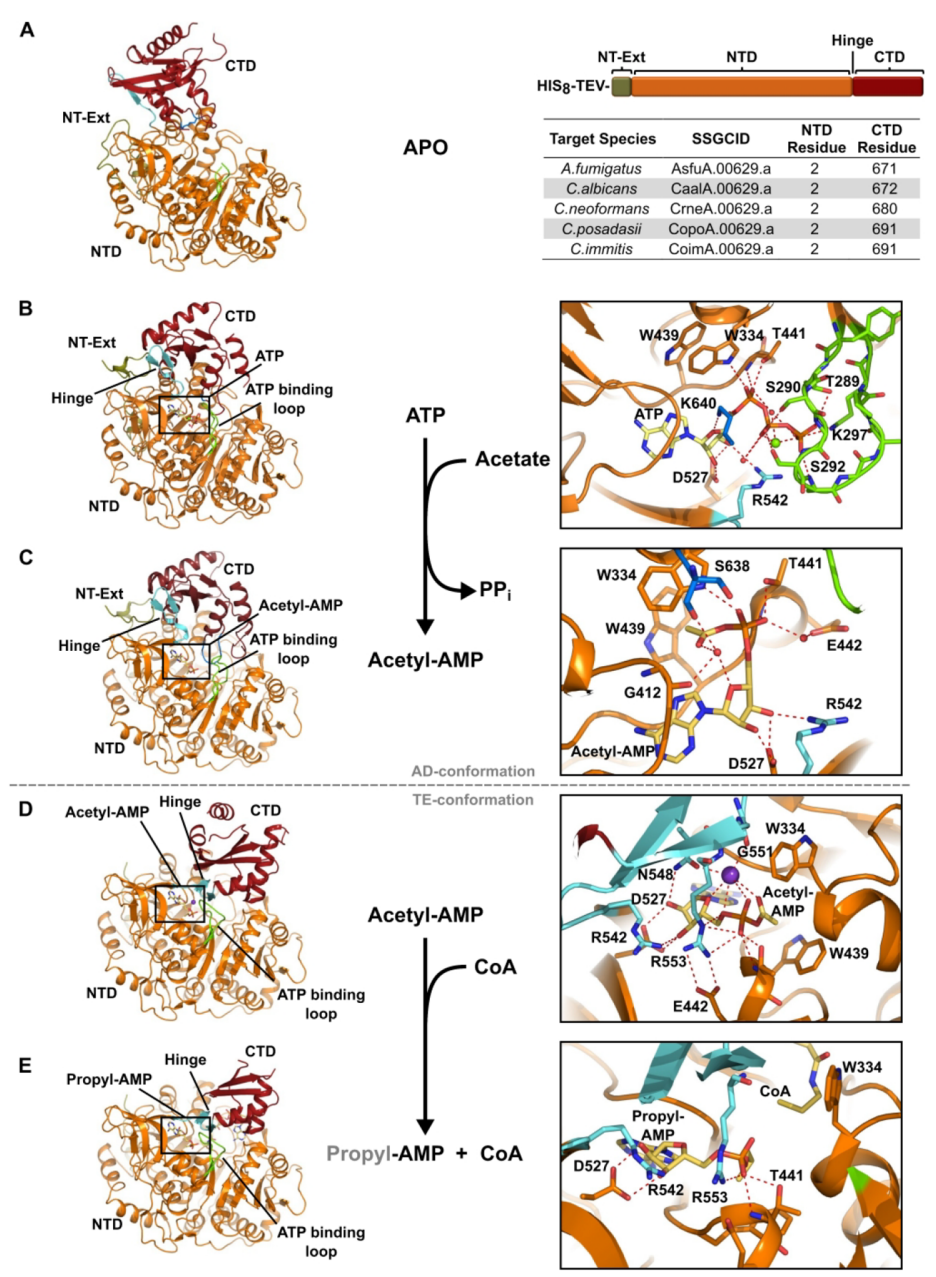
Reaction series for *Cryptococcus neoformans* Acs1. (A) Structure of *Cryptococcus neoformans* Acs1 (PDB 5PVP, chain A, Apo). (B) *Cryptococcus neoformans* Acs1 bound to ATP (PDB 5K8F, chain A, AD-conf); (C) bound to acetyl-AMP, adenylating conformation (PDB 74LG, chain B, AD-conf); (D) bound to acetyl-AMP (PDB 74LG, chain C, TE-conf); and (E) bound to propyl-AMP and coenzyme A, thioesterification conformation (PDB 5K85, chain C, TE-conf). Protein model with table for NTD and CTD start residues of other fungal Acs1 proteins. N-terminal domain (NTD, orange), C-terminal domain (CTD, dark red), N-terminal extension (NT-Ext, olive green), hinge (cyan), ATP binding loop (chartreuse), Lys640 in A (blue), potassium (purple), and ligand (yellow).

With the *Cn*Acs1 crystallization system, we had the opportunity to obtain several structures along the enzymatic pathway from within a single species and mostly within the same crystal form. These include (1) the apo presubstrate-bound form acetylated at the critical active site Lys640, (2) ATP bound (AD-conf), (3) acetyl-AMP (AD-conf product), (4) acetyl-AMP (TE-conf substrate), and (5) the propyl-AMP ester with CoA in the TE-conf. To our knowledge, this is the first structure of an unliganded ACS enzyme, although other ANL family enzymes have been characterized structurally without a ligand.^16^ In the pre-substrate-bound conformation, the CTD adopts unique positions relative to the positions required for adenylation and thioesterification (Figures 3A, S4). Again, *Cn*Acs1 preferred to crystallize as a crystallographic trimer with three copies in the asymmetric unit. The third copy showed no density for the CTD and is expected to be mobile within the neighboring solvent channel. We were able to build the CTD into the first two copies, both in unique positions. In chain A, the CTD is skewed significantly toward the NT-Ext by ∼13 Å with a ∼60° rotation such that β24 is partially melted. This effectively removes Arg542 from the active site where it would be involved with stabilizing the ribose 3′ OH of ATP. The remainder of the active site contributed by the NTD remains ready to bind ATP including a partially ordered ATP binding loop (aa 289–298).

We also observed that the active site lysine (Lys640) was acetylated, likely by *E. coli* acetyltransferases, and was positioned adjacent to the ATP binding site (Figure S8T). The acetylated Lys640 form of the enzyme is unable to catalyze the adenylation reaction,^14^ but this modification does not appear to greatly affect the overall structure of the ATP binding site. A crystal structure of the *S. enterica* Acs acetylated on the corresponding lysine has been reported with a propyl-AMP and CoASH bound, and the authors did not report significant changes from the analogous structure for the unacetylated protein.^22^ In chain B, the CTD is disassociated from the NTD such that β24 and the hinge are fully extended with a gap of ∼12 Å between domains. On the basis of the structures alone, it remains unclear whether either CTD orientation is functionally relevant, but it does suggest that in the absence of a substrate, there is significant flexibility between domains which could help facilitate access of first reaction substrates to the active site.

The ATP-bound structure was achieved through cocrystallization with ATP and magnesium sulfate. Under similar crystallization conditions, *S. cerevisiae* Acs1 crystallized with AMP in the active site rather than ATP.^21^ In contrast, we were pleasantly surprised to see partial occupancy for both ATP and the acetyl-AMP product evidenced by overlapping electron density. Unlike other *Cn*Acs1 structures, all three copies of the enzyme were found in the AD-conf. The α-phosphate is stabilized by interactions with Trp334 and Thr441 as observed with propyl-AMP inhibitor. The β- and γ-phosphates are stabilized by numerous interactions with the ATP binding loop (aa 289–298), which is ordered when bound to ATP (Figure 3B). Interestingly, in this ATP-bound structure, the active site Lys640 coordinates with the ribose ring oxygen and the connecting ribose-phosphate oxygen. Consistent with the previously reported ATP-bound structure of the ANL-family, human medium chain acyl CoA synthetase ACSM2A,^25,26^ we observed density for a single magnesium atom coordinated between the β- and γ-phosphate oxygen atoms. While the electron density in the *Cn*Acs1 structure is weaker due to partial occupancy of ATP and acetyl-AMP, the position of the magnesium and orientation of the ATP-binding loop is consistent with the ACSM2A structure (Figure S5). The Ser290 and Ser292 side-chain hydroxyls hydrogen bond to the β-phosphate, while Thr289, Gly291, Ser292, Thr293, and Lys297 hydrogen bond to the γ-phosphate. The numerous interactions between the ATP binding loop and the β–γ phosphates are consistent with the proposed mechanism for nucleophilic displacement of pyrophosphate during the adenylation half-reaction.

In addition to the mixed ATP/acetyl-AMP bound structure, we also solved a fully occupied acetyl-AMP structure under the same coincubation methods. Importantly, clear density for acetyl-AMP was present in both the AD- and TE-conf; we are unaware of any previous structure showing an adenylated-carboxylate in both reaction conformations. In the AD-conf, the catalytic Lys640 no longer coordinates directly with the adenylated reaction product (Figure 3C). The acetyl-AMP product is rotated and places the methyl group of the adduct into the acyl binding pocket in preparation for the thioesterification reaction. The α-phosphate forms the previously discussed interactions with Trp334 and Thr441 as well as a water-mediated hydrogen bond to Glu442. The acetate adduct is oriented orthogonal to the Trp439 aromatic side chain to form an energetically favorable C–H−π stacking interaction. This arrangement also orients the carbonyl oxygen of the acetyl group toward the ribosyl-phosphate portion of the adenylate. In this way, the acetyl carbonyl, the ribose ring oxygen, the phosphate, and the carbonyl from Gly412 form an interacting array. At the center of this arrangement in the AD-conf, we have modeled a water molecule for chains A and B.

We also observed an acetyl-AMP bound form of the enzyme in the TE-conf. In this case, a potassium ion was modeled in the active site (Figure 3D). The 140° rotation of the CTD also brings additional chelating atoms from the hinge region. Importantly, the position of acetyl-AMP remains static within the AD- and TE-conf of the CTD; in addition to interactions with the potassium ion, the TE-conf establishes new interactions between the acetyl AMP including Asn548 with the ribose 2′ OH, Gly551 with the potassium ion, and Arg553 with both the phosphate and Glu442 (Figure 3D). The observation of the acetyl-AMP adenylation product in both AD- and TE-conf indicates that the conformational change occurs prior to interacting with CoASH and is not induced by CoASH binding.

The TE-conf establishes two important interactions that are likely to facilitate the thioesterification reaction. First, Arg553 interacts with the acetyl-adenylate phosphate group, which is likely to stabilize the developing negative charge as the AMP leaving group is displaced by CoASH. Second, the Trp334 side chain, which was blocking the tunnel through which the pantetheine approaches in the AD-conf,^16^ moves away to reveal the CoA binding site adjacent to the acetate adduct. Consistent with this model, the final snapshot in the enzymatic pathway is *Cn*Acs1 bound to propyl-AMP and CoA in the TE-conf (Figure 3E). Here, propyl-AMP functions as a nonreactive mimic of the acetyl-AMP natural substrate with juxtaposition of the propyl group to the activated thiol of coenzyme A, again with Trp334 rotated away from the phosphate to provide a clear trajectory for the pantetheine to undergo nucleophilic attack on the carbonyl of the acetyl-AMP intermediate. The structural features of this final aspect of the reaction are completely consistent with previous reports of *S. enterica* ACS structures bound to the same ligands.^22^

### Structure–Activity Relationships of Fungal ACS Substrates and Inhibitors

**Figure 4:**
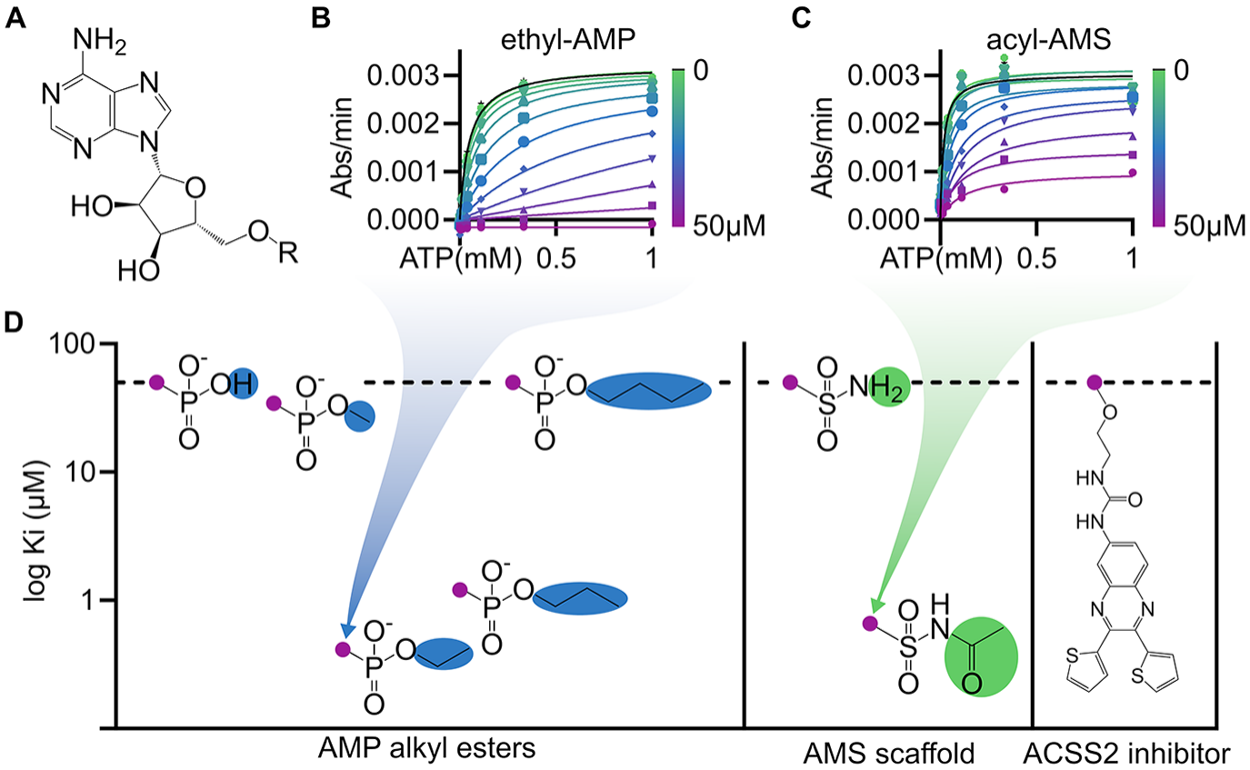
Bisubstrate inhibitors exhibit submicromolar potency. (A) Structure of the adenosine moiety used for bisubstrate phosphodiester or sulphonamide derivatives. (B, C) Representative *Cn*Acs1 enzyme inhibition kinetics of lead compounds display submicromolar *K*_i_ competitive against ATP. (D) SAR of AMP alkyl esters and AMS derivatives for *Cn*Acs1 plotted for potency (*K*_i_) against ATP. Compounds with inhibition above the highest tested concentration are plotted at 50 μM as designated by the horizontal dashed line. No enzyme inhibition was detected by the human ACSS2 inhibitor (VY-3-249).

As discussed in the Introduction, the inhibition of eukaryotic ACSs has emerged as a potential approach to the treatment of a variety of diseases including fungal infections. In addition, the inhibition of other ANL-family enzymes has also been pursued for the treatment of bacterial infections including antibiotic resistant-Gram negative bacteria^27^ and tuberculosis.^28^ One of the most successful approaches to the inhibition of ANL-family enzymes is based on the concept of a bisubstrate inhibitor (Figure 4A).^27−29^ For example, the propyl-AMP ester used in the structural characterization of ACS here, as well as in other studies,^22^ is an example of a bisubstrate inhibitor with the propyl group corresponding to the acetate and the AMP to the adenosine monophosphate; together they mimic the Ac-AMP intermediate. As such, bisubstrate inhibitors are useful as chemical biologic probes that can be used to explore mechanisms of specificity and inhibition. A detailed understanding of the mechanism of the exquisite specificity of ACS will help inform the systematic design of inhibitors. To do so, we tested the activity of a series of alkyl-AMP bisubstrate inhibitors, an acylsulfamate bisubstrate inhibitor, and a recently reported non-nucleoside-based inhibitor of human ACSS2.^5^

**Figure 5:**
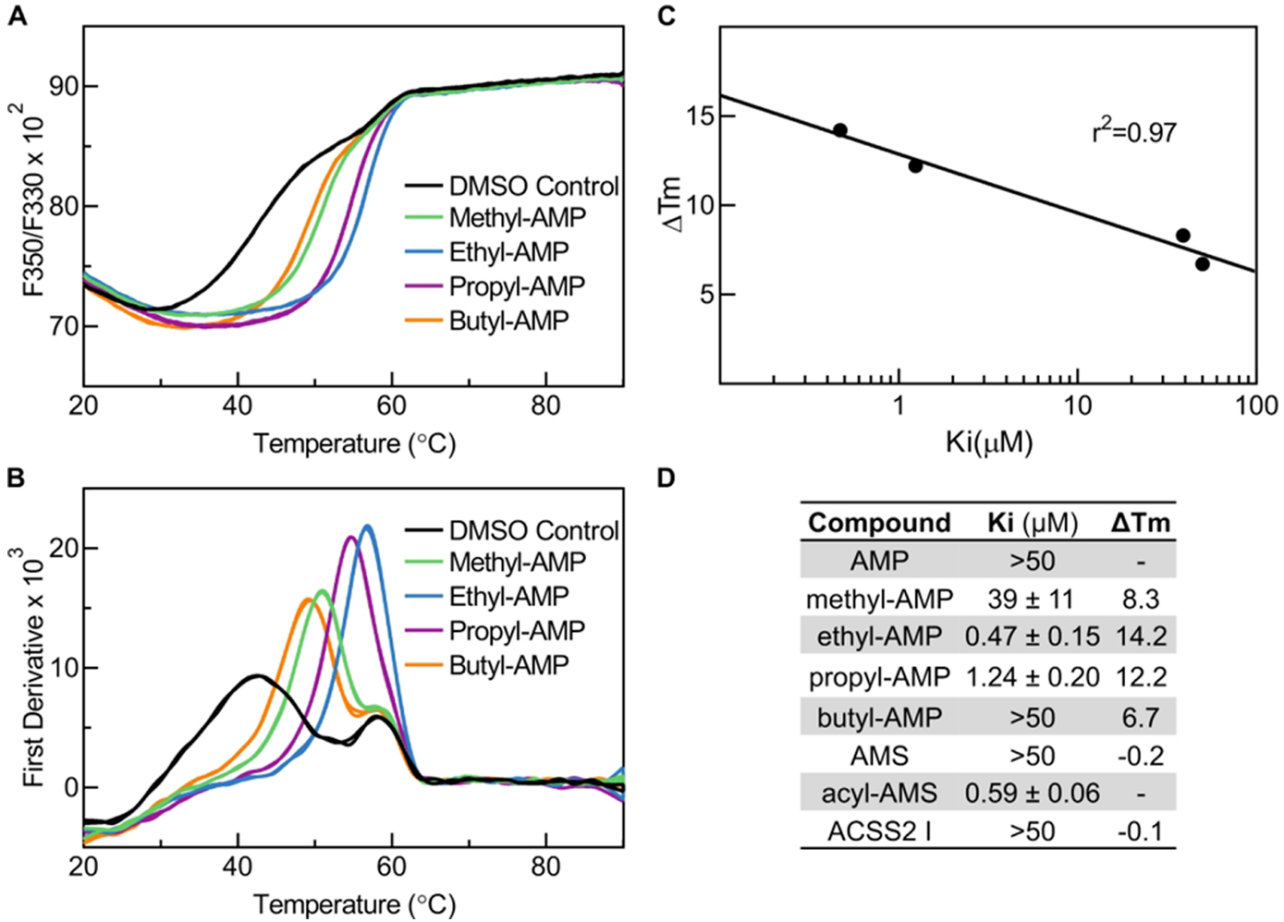
Thermal shift induced by alkyl AMP esters tightly correlates with inhibitor potency. (A) Thermal profile of fluorescence ratio F350/F330. (B) Thermal profile of the first derivative. The traces in panel A were smoothed using the smoothing function of GraphPad Prism. The *T*_m_ of the protein is represented by the peaks in panel B. (C) Correlation of thermal shift from DMSO control with inhibitor potency. (D) Table of inhibitor potency (*K*_i_) and thermal shift (Δ*T*_m_).

The importance of the bisubstrate nature of the alkyl-AMP inhibitors is illustrated by the fact that the reaction product, AMP, is a weak inhibitor of ACSs as reported elsewhere; we confirmed that to be the case for *Cn*Acs1 (*K*_i_ > 50 μM, Figure 5D). Grayson and Westkaemper^30^ first reported that the conversion of the AMP to an alkyl ester dramatically increases its potency as an ACS inhibitor (Figures 4D, 5D). The alkyl AMP esters are competitive with ATP, and nanomolar *K*_i_ values were observed for both the ethyl and propyl AMP esters with the ethyl ester showing the lowest apparent *K*_i_ of 470 nM (Figures 4D, 5D). The methyl ester, on the other hand, was ∼100-fold less active than ethyl, while the butyl ester showed no inhibition at the limit of solubility. The structure–activity relationship shown by the AMP ester for enzyme inhibition is also observed by thermal shift assays of inhibitor-target engagement. As shown in Figure 5, the complexes of Acs1 with methyl and butyl esters showed significantly lower Δ*T*_m_ values than the complexes with ethyl and propyl esters. This indicates that the variations in inhibitor potency correlate with direct interactions with *Cn*Acs1, which is also demonstrated by the correlation between IC_50_ and Δ*T*_m_.

In keeping with the concept of the bisubstrate inhibitor mechanism of these inhibitors, we also examined the activity of acetate, propionate, butyrate, and valerate as substrates for *Cn*Acs1. Once more, acetate was the optimum substrate with propionate much poorer with a *K*_m_ > 100-fold higher; neither valerate nor butyrate were *Cn*Acs1 substrates (Figure S6). These data clearly demonstrate the highly selective nature of the alkyl acid binding interactions in ACS enzymes and are consistent with previous characterizations of Acs substrate specificity.^19^ In terms of size, the ethyl group best approximates the acetyl moiety of the AMP-Ac intermediate. The extremely steep nature of the AMP-ester SAR and the enzyme substrate specificity clearly demonstrates the importance of the contacts established by the properly sized small alkyl group as well as the specificity of the binding pocket that accommodates those contacts. We also compared the activity of the ethyl- and butyl-AMP derivatives across the different human fungal pathogen ACSs and found that they were similarly active (Table S4).

Alkyl AMP esters are quite toxic and, therefore, do not represent viable inhibitors in biological systems.^29^ To address these issues and to generate more stable inhibitors, sulfonamide isosteres of the phosphate group have been developed in the context of other ANL-family enzyme inhibitors.^27−29^ To further explore the activity of stabilized mimics of the AMP-Ac intermediate, we tested the acetyl-**a**denosine **m**ono**s**ulfonamide (Ac-AMS) bisubstrate inhibitor generously provided by Dr. Courtney Aldrich (Figure 4D). Ac-AMS inhibited *Cn*Acs1 with a *K*_i_ of 0.59 μM in an ATP competitive manner (Figure 4 C,D). In contrast, the glycine derivative of AMS gave no inhibition (data not shown), suggesting that positive charge is not tolerated in the binding pocket. Interestingly, bisubstrate inhibitors derived from AMS for other adenylating enzymes were much more potent and showed tight-binding characteristics.^29^ Due to the detection limit of our assay and reported *K*_i_’s approaching enzyme concentrations in the reaction, we only report apparent *K*_i_’s. As a benchmark, we calculated the binding energy of a bisubstrate inhibitor taking full advantage of ATP (Δ*G* = −6 kcal/mol, *Cn*Acs1) and acetate (Δ*G* = −5.5 kcal/mol, *Cn*ACS) binding with an expected total binding of Δ*G* = −11.5, which would correspond to a *K*_i_ of 7.8 nM for *Cn*ACS. These calculations assume full activity from our enzyme preps and recognize that a bisubstrate inhibitor may bind more tightly than the energies of the individual substrates, but linking of the substrates may also result in a loss of interaction with the enzyme.^31^ Nonetheless, the reported potencies of these inhibitors are likely conservative given that the apparent *K*_i_’s are approximately half the concentration of the enzyme tested, clearly supporting a tight-binding mode of inhibition. Unfortunately, none of the inhibitors had antifungal activity, likely due to the fact that their negative charge significantly reduces penetration of the fungal cell wall.

Finally, Comerford et al. report the identification of an inhibitor of human ACSS2 enzyme (VY-3-249) from a high throughput screen (Figure 4D).^4^ Alignment of ACSS2 and *Cn*Acs1 indicated that there are regions of conservation, particularly in the core domains found across all ACSs; however, there is evidence of reasonable divergence with the proteins showing 44% sequence identify. Interestingly, VY-3-249 showed no activity against the *Cn*Acs1 up to 100 μM; higher concentrations showed interference with the assay. Thermal shift assays also showed no evidence that VY-3-249 interacted with the *Cn*Acs1 (data not shown). We used the same type of biochemical ACS activity assay as Comerford et al.,^4^ who found that VY-3-249 inhibited ACCS2 with an IC_50_ of 5–8 μM. At this point, it is unclear why the inhibitor is not active against *Cn*Acs1.

Although it is tempting to suggest that there may be specificity differences between the human and fungal enzymes, the residues that comprise the activity sites of the enzymes are well-conserved. Recently, Miller et al. reported that this inhibitor inhibited ACSS2 at less than 50% at the highest drug concentrations, suggesting that it is not a particularly effective inhibitor of ACS.^34^ Thus, small differences in affinity between the two enzymes for the inhibitor may be amplified. Since ACSS2 does not appear essential in mammals,^4,5^ high specificity for fungal enzymes, however, is not likely to be as important as potent on-target activity in the development of antifungal ACS inhibitors.

### Bisubstrate Inhibitor Complexes Provide Structural Insights into the Mechanism of ACS Alkyl Acid Substrate Discrimination

**Figure 6:**
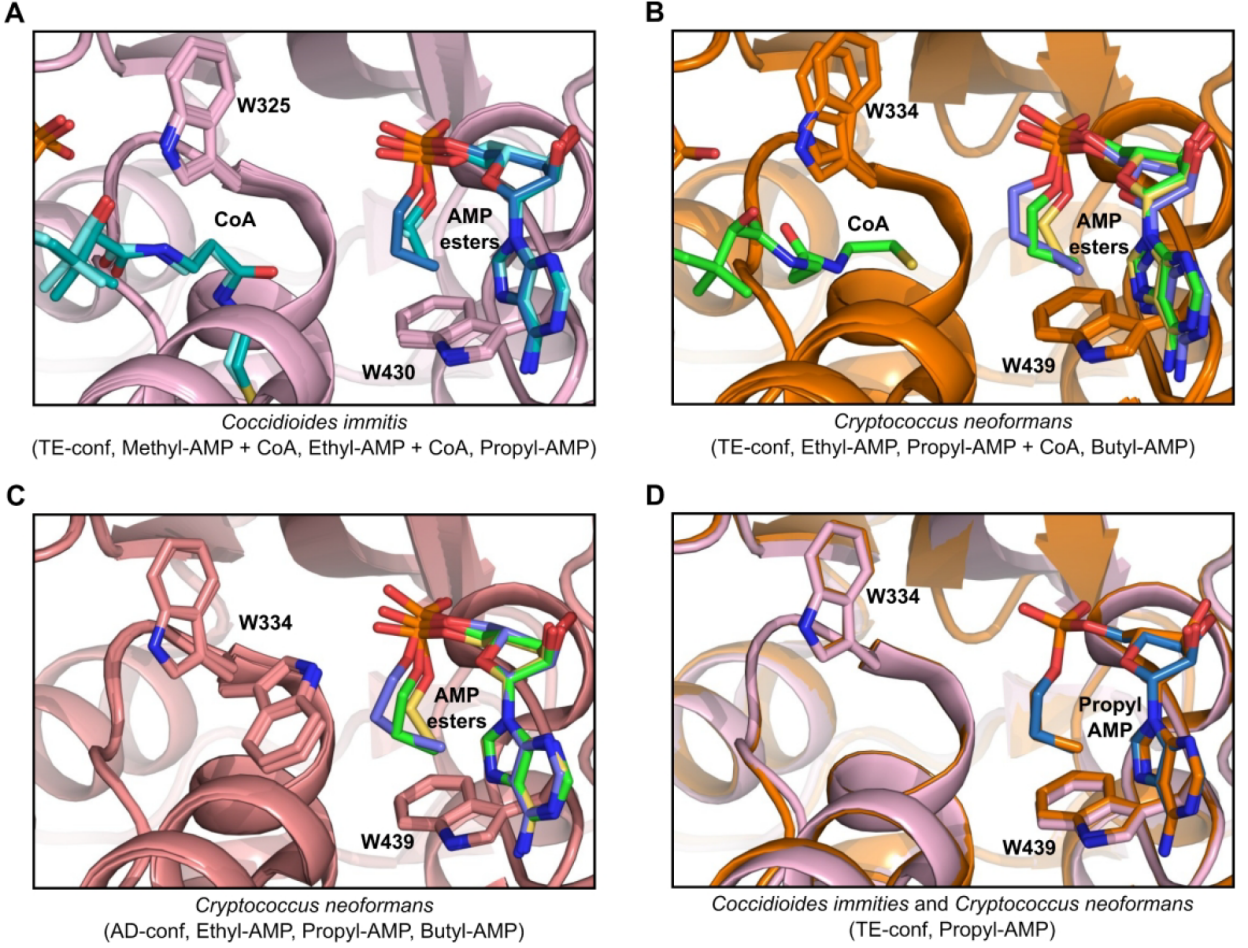
Overlay of AMP ester series bound structures for*Coccidioides immitis* and *Cryptococcus neoformans* Acs1 crystal structures. (A) Overlay of *Coccidioides immitis* Acs1 bound to methyl-AMP + coenzyme A (PDB 7L3Q, aquamarine), ethyl-AMP + coenzyme A (PDB 7KVY, teal), and propyl-AMP (PDB 7KQ6, slate). Protein shown in pink. (B) Overlay of *Cryptococcus neoformans* Acs1 bound to ethyl-AMP (PDB 7KNO, yellow), propyl-AMP + coenzyme A (PDB 5K85, green), and butyl-AMP (PDB 7KNP, blue) in the thioesterification conformation (TE-conf). Protein shown in orange. (C) Overlay of *Cryptococcus neoformans* Acs1 bound to ethyl-AMP (yellow), propyl-AMP + coenzyme A (green), and butyl-AMP (blue) in the thioesterification conformation (AD-conf). Protein shown in salmon. (D) Overlay of *Coccidioides immitis* (pink, protein; blue, compound) and *Cryptococcus neoformans* (protein and compound, orange) propyl-AMP bound crystal structures.

To understand the structural basis for the ability of ACS to discriminate between alkyl-AMP inhibitors, and by extension alkyl carboxylic acid substrates, we sought to crystallize ACS with the range of alkyl-AMP inhibitors in the presence or absence of CoA in both the *Cryptococcus* and *Coccidioides* Acs1. Methyl-AMP and ethyl-AMP were crystallized with *Coccidioides* Acs1 with, and without, CoA and with propyl-AMP alone; CnAcs1 was crystallized with propyl-AMP, with and without coenzyme A, and with ethyl-AMP and butyl-AMP alone. Again, the only difference in the active site is Met440 in *Cryptococcus* Acs1 and glutamine in *Coccidioides* Acs1. Overlays of the AMP ester series are shown in Figure 6. All *Coccidioides* cocrystal structures were solved in the TE-conf such that Trp325 (equivalent to *Cryptococcus* Trp334) is swung out of the active site to allow CoA access to the AMP ester pocket. In the *Coccidioides* bound series of methyl, ethyl, and propyl-AMP inhibitors, as in the *Cryptococcus* bound series of ethyl, propyl, and butyl-AMP inhibitors, we observed the increasing longer aliphatic chains in roughly equivalent positions within the active site (Figure 6A). The ethyl-AMP is aligned in a conformation that places the alkyl group nearly orthogonal (3.5 Å, 153°) to the Trp439 (equivalent to *Coccidioides* Trp430) aromatic ring (Figure S7A). The −OCH_2_–CH_3_ is found in a low-energy staggered confirmation that places the terminal methyl group in position for a favorable C–H−π stacking interaction with the aromatic portion of Trp439. The methyl-AMP overlaid well with the ethyl-AMP methylene, but the distance between the methyl group and the aromatic ring is larger than with the ethyl-AMP and, thus, likely reduces the strength of that interaction.

In contrast, the propyl-AMP and butyl-AMP alkyl chains adopt a sterically unfavored conformation. The terminal CH_3_ group of the propyl-AMP and butyl-AMP overlaps with the terminal CH_3_ of the ethyl-AMP and, thus, maintains VDW distance from the Trp439 aromatic ring (3.4 Å, 3.6 Å, respectively); however, the optimal C–H−π stacking angle is lost (174° observed for acetyl-AMP versus 118° for propyl-AMP or 86° for butyl-AMP; Figure S7B–D). Therefore, unlike the energetically favorable anticonformation of the ethyl-AMP alkyl group, the ACS bound butyl-AMP is in a very high energy conformation (Figure S7E). The butyl-AMP has a dihedral angle of 41.6° (OCCC) between the third carbon of the butyl side chain and the phosphate oxygen atom when bound. This conformation is 1.70 kcal/mol higher than the lowest energy conformation, which would have an anticonformation. Additionally, the dihedral angle between C1 and C4 of the butyl group is 63.2°. This nearly gauche interaction is 0.53 kcal/mol higher than the relative anticonformation. Relative energy calculations were performed using MM2 generated with Chem3D software. The higher energy conformation adopted by the butyl-AMPs likely contributes to its reduced ability to stabilize the ACS enzyme, its lower potency as an inhibitor relative to the ethyl-AMP, and the lack of reactivity of similarly sized carboxylic acid substrates (Figures 4, S6). These structures provide strong support for the concept that the Trp439 residue functions as a key mechanism that limits the size of the alkyl carboxylic acids accommodated in the active site and structurally confirm models previously developed based on the effects of replacing Trp439 with glycine.^16,19^

**Figure 7:**
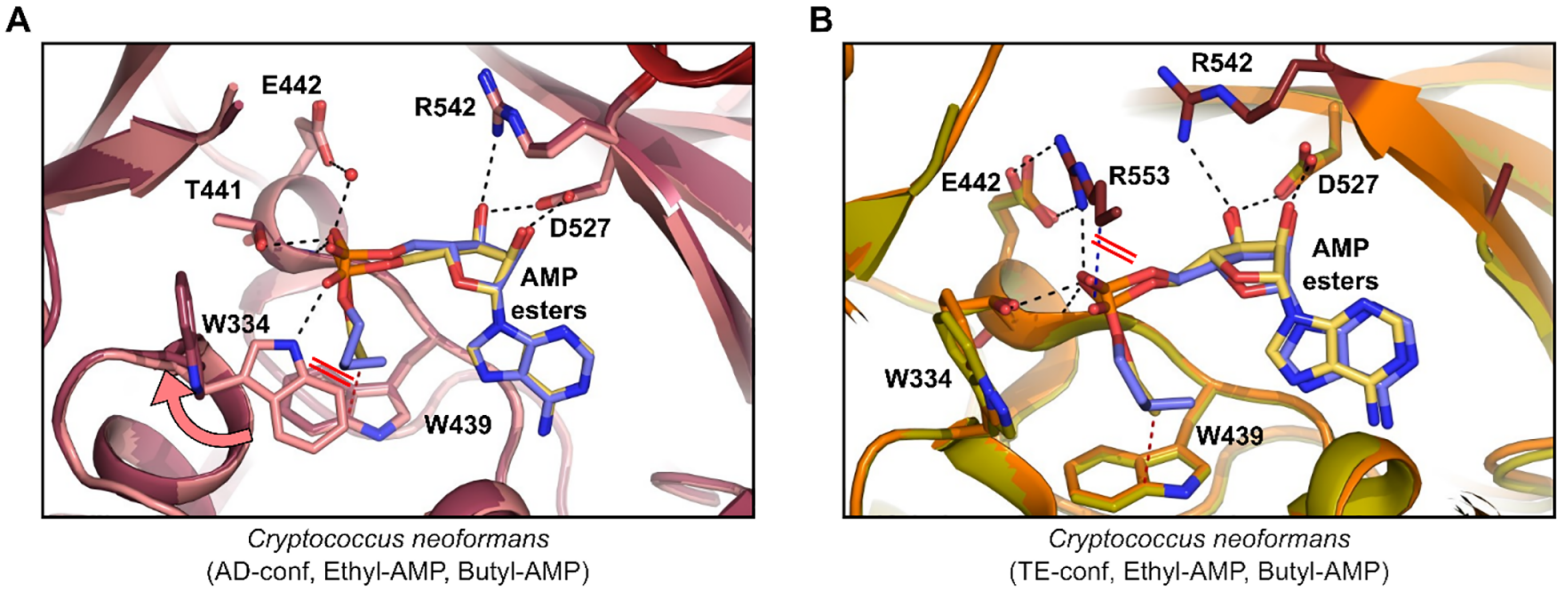
Comparison of *Cryptococcus neoformans* Acs1 ethyl and butyl-AMP inhibitor bound structures in the AD and TE conformations. (A) Overlay of ethyl-AMP (PDB 7KNO; compound, yellow; protein, pink) and butyl-AMP (PDB 7KNP; compound, blue; protein, dark pink) in the AD-conf. (B) Overlay of ethyl-AMP (PDB 7KNO; compound, yellow; protein, orange) and butyl-AMP (PDB 7KNP, compound, blue; protein, green) in the TE-conf. Red lines indicate new clashes observed.

As the aliphatic chain length increases in this series and pushes against Trp439, this interaction leads to a progressively larger displacement of the phosphate from the active site; specifically, the Trp334 indole NH to PO_4_ distance increases 2.7 Å, 3.3 Å, and 3.9 Å in the ethyl, propyl, and butyl AMP structures, respectively. This trend is observed in both the AD- and TE-conf. With *Cn*Acs1 in the AD-conf, as previously discussed, the propyl-AMP bound structure showed Trp334 in two rotamer conformations. One conformation involves a hydrogen bond between the Trp334 indole NH and the AMP phosphate and a second where the indole side chain is displaced from the binding site (consistent with the TE-conf). In the ethyl-AMP bound form, the Trp334 is only found oriented toward the compound, while in the longest length butyl-AMP bound form, the Trp334 side chain is only found away from the active site. Upon further examination, due to the high energy conformation of the aliphatic chain, the methylene groups for both propyl and butyl-AMP inhibitors clash with the Trp334 aromatic side chain (ethyl, 3.7 Å; propyl, 3.2 Å; butyl, 2.3 Å, Figure 7A). Together, this leads to displacement of Trp334 from interaction with the AMP phosphate.

A comparison of the acetyl-AMP bound with structures with the two mimics of this intermediate, ethyl-AMP and acetyl-AMS, confirms that the inhibitors interact with the enzyme in a manner very much like the product of the adenylation reaction. The electronic and functional group differences between the alkyl AMP ester, the acetyl-sulfonamide, and the acetyl-AMP reaction product do not appear to have a significant effect on the ability of these three molecules to bind within the active site of the enzyme. Consistent with that conclusion, the ethyl-AMP and acetyl-AMS inhibitors have similar IC_50_ values for *Cn*Acs1. A variety of chemically distinct isosteres of nucleoside phosphates have been applied to the design of inhibitors.^32,33^ It appears that the ACS active site might offer flexibility at this position and accommodate chemically distinct isosteres. The placement of an alkyl group of the proper size appears to be much more important for binding than the electronic features of the groups connecting the alkyl group to the adenosine moiety.

An important contribution that our study provides is a characterization of the structural consequences of inhibitors with different sizes of the alkyl substituents. Previous structures for ACS enzymes had employed the propyl-AMP ester to facilitate crystallization. We were able to obtain structures for methyl-, ethyl-, propyl-, and butyl-AMP esters, a set of inhibitors with very different abilities to bind and inhibit ACS enzymes (Figures 4, 5C). At first glance, it would seem quite remarkable that a structure could be obtained for a poor inhibitor such as butyl-AMP. It is important to recall that the first structure for an ACS enzyme was the *S. cerevisiae* Acs1 bound to AMP,^20^ which is also a quite poor ligand and inhibitor of the enzyme. In addition, thermal shift assays indicate that high concentrations of these poor inhibitors are able to stabilize the enzyme relative to the apo-form of the enzyme.

The determinants of the specificity of ACS for the shortest chain alkyl carboxylic acid, acetate, was probed genetically by Ingram-Smith et al. using the *M. thermautotrophicus* enzyme.^19^ They showed that mutation of the residue corresponding to Trp439 (*Cn*Acs1 numbering; Trp416 in MtAcs) to the much smaller glycine dramatically affected the substrate specificity of the enzyme and allowed alkyl groups up to seven carbons as well as branched chain acids to be utilized as substrates. These biochemical data supported a previous hypothesis by Gulick that acyl-CoA synthetases with Trp at this position indicated specificity for small carboxylic acids such as acetate.^16^ Molecular modeling of the MtAcs enzyme containing the Gly416 mutation further supported the conclusion that the Trp residue was a key determinant of the size of the alkyl group that could productively bind to the active site.^19^ The structure activity and structural analysis of a range of alkyl-AMP esters provide additional mechanistic support for that model.

The most remarkable structures are those of the butyl-AMP ester bound to both adenylation (AD) and thioesterifcation (TE) conformations of *Cn*Acs1. The terminal methyl group of the butyl moiety overlaps with the terminal methyl groups of the smaller alkyl esters with the distance to the aromatic ring of the Trp439 indole ring maintained between 3.4 and 3.6 Å. The butyl group in the TE-conf, however, is contorted into an extremely high energy conformation that includes a nearly eclipsed interaction as well as a gauche conformation and loss of the stabilizing C–H−π bond. Thus, the Trp remains essentially unchanged relative to its position when bound to more favorably sized ligands. These data provide extremely strong structural support for the Trp wall model as the mechanism for limiting substrates to small carboxylic acids in ACS enzymes. It also may be the case that the Trp functions not just as a wall but also providing favorable interactions through C–H−π interactions. The less potent inhibitor, methyl-AMP, places the methyl group farther away from the Trp439 indole ring and thus does not have optimal stabilizing interactions with the aromatic ring.

In addition to poor binding features of the butyl-AMP ester in the adenylating reaction, we also observe similar disruption of key interactions in the TE-conf (Figure 7B). In this conformation, the CTD of the *Cryptococcus* Acs1 structure is disordered. Overlay of the ethyl-AMP structure on the butyl-AMP structure gives a potential explanation. In the TE-conf, Arg553 forms two salt bridges to Glu442 and a single hydrogen bond to the ethyl-AMP phosphate. Displacement of the phosphate due to the longer aliphatic chain of butyl-AMP pushes the phosphate such that the free oxygen which coordinates with Trp334 in the AD-conf would now clash with the aliphatic portion of the Arg553 side chain. Loss of this interaction may lead to destabilization of the CTD position over the active site. Electron density for butyl-AMP in the TE-conf was of lower quality than the AD-conf such that the compound was modeled at 86% occupancy in the active site. Therefore, the optimal inhibitor is the one which best mimics the acetyl-AMP intermediate bound state, which is the ethyl-AMP ester inhibited complex (Supplemental Figure S8).

The acetyl-AMS inhibitor and the ethyl-AMP have similar potency. We, therefore, sought to compare the structures of the ACS bound to those two inhibitors. We successfully crystallized *Cn*Acs1 bound to acetyl-AMS. All three copies present in the asymmetric unit were in the AD-conf. The acetyl-AMS inhibitor shares many structural and electronic features with the acetyl-AMP reaction intermediate. Consistent with those similarities, the structures for the acetyl-AMS and the acetyl-AMP bound enzymes are essentially identical. The active sites overlay well, and hydrogen bonds to Trp334 and Thr411 are maintained. The orientation of the acetate moiety of the inhibitor is orthogonal to the plane of the Trp334 aromatic side chain. The position of the acetyl methyl group overlaps well with the methyl groups of both the actual reaction intermediate and the methyl group of the ethyl-AMP inhibitor (Supplemental Figure 9). Interestingly, the structural and biochemical similarities of the acetyl-AMS and ethyl-AMP inhibitors suggest that the carbonyl group of the acetyl group does not contribute significantly to the binding of the substrate or inhibitors, suggesting that this may be a position that can be modified in the design of new inhibitors.

In summary, we have carried out an extensive characterization of fungal ACS enzymes using biochemical, chemical biology, and X-ray structural analyses. These studies have provided detailed mechanistic information regarding both the reaction mechanism and the structural basis for substrate specificity. As interest in targeting this class of enzymes with drugs increases, these data should be useful for the design of molecules that target not only fungal ACSs but other eukaryotic enzymes as well.

## Methods

### Cloning of Expression Constructs

Full length *ACS1* genes for species *Aspergillus fumigatus* (AsfuA.00629.a, Uniprot Q4WQ02 2–670), *Coccidioides immitis* (CoimA.00629.a, Uniprot A0A6C1M7V3 2–691), *Coccidioides posadasii* (CopoA.00629.a, Uniprot C5PGB4 2–691), *Candida albicans* (CaalA.00629.a, Uniprot Q8NJN3 2–671), and *Cryptococcus neoformans* (CrneC.00629.a, Uniprot J9VFT1 2–680) with the N-term 8×His-Tev tag (MHHHHHHHHENLYFQG) were codon-optimized using ATUM for *E. coli* expression and cloned by ATUM into ATUM vector pD431-SR via SapI cloning, including a double stop after the open reading frame (ORF). An example of the 5′ adapter just prior to ATG is 5′-TACACGTACTTAGTCGCTGAAGCTCTTCT-3′ and the 3′ adapter just after the double stop is 5′-TAGGTACGAACTCGATTGACGGCTCTTCTACC-3′. Codon-optimization excluded restriction sites NcoI, NdeI, XhoI, *Hin*dIII, and SapI. The pD431-SR vector is kanamycin-resistant with the p15a origin of replication accepting inserts under the T7 promoter with a lac repressor and strong ribosome binding site (RBS). The resulting plasmids were sequence verified and transformed into BL21(DE3) (NEB C2527) prior to expression studies.

### Recombinant Expression and Purification

*ACS1* constructs were transformed into BL21(DE3) *Escherichia coli* cells (New England Biolabs). A starter culture grown in Terrific Broth medium (Sigma-Aldrich) containing 50 μg/mL kanamycin (Teknova) was inoculated and grown at 37 °C overnight. The following day, the starter culture was used to inoculate the large-scale culture (4–8 L). At an OD_600_nm of 0.5–0.55, the large-scale culture was transferred from 37 to 25 °C and equilibrated for an additional 30 min before inducing expression with 1 mM IPTG (Teknova). The cells were harvested by centrifugation after overnight growth at 25 °C, and the cell pellet was frozen before purification. Cell pastes were thawed and resuspended at a 1:4 weight/volume for 30 min in buffer containing 25 mM Tris at pH 8.0 (Corning), 200 mM NaCl (Teknova), 0.5% glycerol (Sigma-Aldrich), 0.02% CHAPS (VWR), 5 mM imidazole (Sigma-Aldrich), 1 mM TCEP (Soltec Ventures), 50 mM arginine (Sigma-Aldrich), 100 mg of lysozyme (Affymetrix), 500U benzonase (Millipore), and one EDTA-free protease inhibitor (Thermo Scientific). The resuspension was sonicated (70% amplitude, 30 s process time, 2 s on, 1 s off, on ice) and then microfluidized (15 000 PSI, 2 passes on ice) at 4 °C for 30 min. The lysed suspension was then clarified by centrifugation at 142 000 RCF at 4 °C for 30 min. The resulting supernatant was filtered through a 0.2 μm PES bottle top filter (Nalgene) before starting affinity chromatography. The targets were captured by Ni-charged HiTrap chelating chromatography (GE). The charged columns were equilibrated in buffer containing 25 mM Tris at pH 8.0, 200 mM NaCl, 50 mM arginine, 1 mM TCEP, and 0.25% glycerol. After loading the supernatant, the columns were washed with 10 CV of wash/equilibration buffer and eluted over 120 min in a gradient of 0–60% elution buffer containing 25 mM Tris at pH 8.0, 200 mM NaCl, 1 mM TCEP, and 500 mM imidazole. Fractions containing the target were pooled and digested overnight at 4 °C with in-house TEV protease at a ratio of 1:100 protease to target, simultaneous with dialysis into buffer containing 25 mM Tris at pH 8.0, 200 mM NaCl, 1 mM TCEP, and 0.25% glycerol (SnakeSkin dialysis tubing, 10 kDa MWCO). The dialyzed and digested protein solution was applied to a second Ni purification step to remove uncleaved material and impurities. The buffers used matched those from the first Ni purification step. The protein located in the flow-through and early wash fractions of the nickel subtraction step was pooled and concentrated with a Vivaspin PES 30 kDa MWCO spin concentrator (5–6k RCF in 10 min intervals) to 15 mg mL^–1^ for size exclusion chromatography (SEC). SEC was performed using a Sephacryl S-200 column (GE) equilibrated in 10 mM Tris at pH 8.5 and 20 mM NaCl. Fractions containing the target were pooled and concentrated to 20 mg mL^–1^ for crystallography and then flash frozen in liquid nitrogen. Typical yields were up to 10 mg g^–1^ of cell paste.

### Chemical Synthesis of Compounds

Methods and materials for the synthesis of AMP esters and Acyl-AMS are provided in the Supporting Information.

### Enzyme Activity Detection and Michaelis–Menten Kinetics

Enzyme activity was measured spectrophotometrically by monitoring continuous pyrophosphate release linked to accessory enzymes and carried out according to the EnzChek Pyrophosphate Assay Kit (Thermo). 2-Amino-6-mercapto-7-methylpurine riboside (MESG), purine nucleoside phosphorylase (PNP), and pyrophosphatase were all diluted and stored according to the manufacturer’s instructions. Proteins were diluted using 1× reaction buffer provided in a kit (CnACS 33.0 μg mL^–1^, AfACS 7.8 μg mL^–1^, CaACS 4.0 μg mL^–1^, CiACS 8.0 μg mL^–1^, CpACS 0.67 μg mL^–1^). The following reagents were supplemented to the reaction buffer: 4 mM MgCl_2_ and 10 mM DTT. Reaction volume was scaled to 50 μL in a half-well 96-well plate format. The following reaction substrates were supplied in excess when other substrates were varied: ATP 2.5 mM, CoA 1 mM, and sodium acetate 0.5 mM. All reactions were assembled at RT without acetate and then incubated at 37 °C for 10 min to consume any background phosphate. Acetate was then added to start the reaction and continuously monitored at Abs 360 nm and 37 °C in a SpectraMax i3X Multi-Mode plate reader (Molecular Devices). Product formation was determined using a pyrophosphate standard curve following manufacturer instructions scaled to 50 μL reactions. Enzyme kinetics (*K*_m_) for each substrate were determined from the nonlinear regression of the slopes for the respective concentration dilution series (GraphPad Prism). Inhibition constants (*K*_i_) were determined using the nonlinear regression of the apparent *K*_M_ determined across each inhibitor concentration dilution series (GraphPad Prism).

### Nano Differential Scanning Fluorimetry

Purified Acs1 was diluted to 0.5 mg mL^–1^ (6.64 μM) in 10 mM tris at pH 8.5 and 20 mM NaCl. Each compound (methyl-AMP, ethyl-AMP, propyl-AMP, and butyl-AMP) was prepared in 100% DMSO at 25 mM. Samples were made at 4% final DMSO and 1 mM final compound concentrations. All samples were then incubated on ice for 15 min. Each was then loaded into capillaries in triplicate. A temperature gradient of 20 to 90 °C was run at a rate of 1 °C/min with a laser power of 30% at an excitation wavelength of 285 nm, and fluorescence was monitored at 330 and 350 nm. Data collection was performed on the NanoTemper Prometheus NT.48 and analyzed in GraphPad Prism.

## Supporting Information Available

Supplemental Figure 1: recombinantly expressed and purified Acs1 proteins. Supplemental Figure 2: ACS activity assay validation. Supplementary Figure 3: N-terminal Extension (NT-Ext) from *Cryptococcus neoformans* Acs1. Supplemental Figure 4: conformational orientations of the C-terminal domain of *Cryptococcus neoformans* Acs1. Supplemental Figure 5: comparison of Human ACSM2A and *Cryptococcus neoformans* Acs1 bound to ATP and magnesium. Supplemental Figure 6: utilization of alternative acid substrates. Supplemental Figure 7: angle and distances from terminal methyl to Trp439 side chain in *Cryptococcus neoformans* AD conformation. Supplemental Figure 8: comparison of ethyl-AMP and acetyl-AMP bound to *Cryptococcus neoformans* Acs1. Supplemental Figure 9: overlay of acetyl-AMP and acetyl-AMS crystal structures. Supplemental Figure 10: stereo images of substrate binding pockets for fungal Acs1 crystal structures. Supplemental Table 1: crystallographic data and refinement statistics. Supplementary Table 2: structural and sequence comparison of Acs1. Supplemental Table 3: alignment of fungal Acs1. Supplemental Table 4: potency of ethyl-AMP and butyl-AMP across fungal Acs1 recombinant proteins. Synthetic procedures for the preparation of AMP alkyl esters, AMS, and AcAMS. Crystallization conditions and procedures. Structure determination (PDF)

## Abbreviations

ACS: acetyl CoA synthetases
AD-conf: adenylation confirmation
AMP: adenosine monophosphate
AMS: adenosine monosulfonamide
ANL: acyl-CoA/NRPS/luciferase
ATP: adenosine triphosphate
CoA: coenzyme A
CTD: C-terminal domain
IMAC: immobilized metal affinity chromatography
MESG: 7-methyl-6-thioguanosine
NTD: N-terminal domain
NT-Ext: N-terminal extension
PDH: pyruvate dehydrogenase
SAR: structure–activity relationship
SEC: size exclusion chromatography
TE-conf: thioesterification confirmation
