# Supplementary material for: Structural Characterization of the Reaction and Substrate Specificity Mechanisms of Pathogenic Fungal Acetyl-CoA Synthetases
Source: ACS Chem Biol. 2021 Aug 9;16(8):1587–99. doi: 10.1021/acschembio.1c00484 (PMC8383264; doi:10.1021/acschembio.1c00484)
Supplement: Supplementary file 1 — cb1c00484_si_001.pdf [file cb1c00484_si_001.pdf]

Structural characterization of the reaction and substrate specificity mechanisms of pathogenic fungal acetyl-CoA synthetases.

Andrew J. Jezewski[1], Katy M. Alden[1], Taiwo E. Esan[2], Nicholas D. DeBouver[3,5], Jan Abendroth[3,5], Jameson C. Bullen[3,5], Brandy M. Calhoun[3,5], Kristy T. Potts[3,4], Daniel M. Murante[1], Timothy J. Hagen[2], David Fox[3,4,5], and Damian J. Krysan*[1,6]

[1]Department of Pediatrics Carver College of Medicine, University of Iowa, Iowa City, IA 52242

[2]Department of Chemistry and Biochemistry, Northern Illinois University, DeKalb, IL 60115

[3]UCB Pharma., 7869 NE Day Road West, Bainbridge Island, WA 98110

[4]Beryllium Discovery Corp., 7869 NE Day Road West, Bainbridge Island, WA 98110

[5]Seattle Structural Genomics Center for Infectious Disease (SSGCID), Seattle, WA, 98109

[6]Microbiology/Immunology, Carver College of Medicine, University of Iowa, Iowa City, IA 52242

Running title: Substrate specificity of acetyl-CoA synthetases

Corresponding Author:

Damian J. Krysan

2040 Med Labs 25 S. Grand Avenue, Department of Pediatrics and Microbiology/Immunology, Carver College of Medicine, University of Iowa, Iowa City Iowa 52242, Phone: 319-335-3066, damian-krysan@uiowa.edu

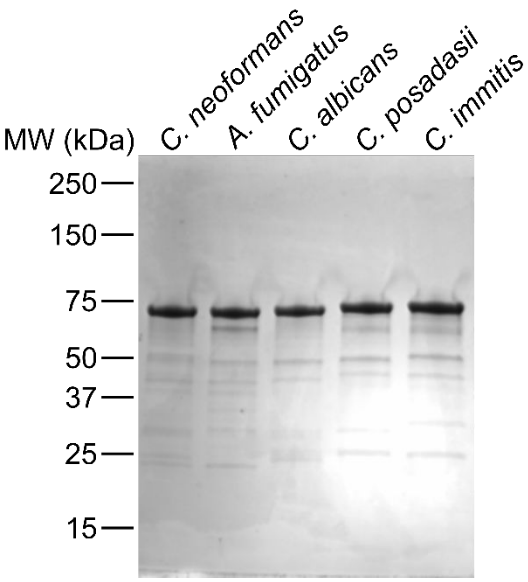

Supplemental Figure 1. Recombinantly expressed and purified Acs1 proteins. Coomassie gel represents 2ug of loaded purified proteins with dominant bands representing >95% purity.

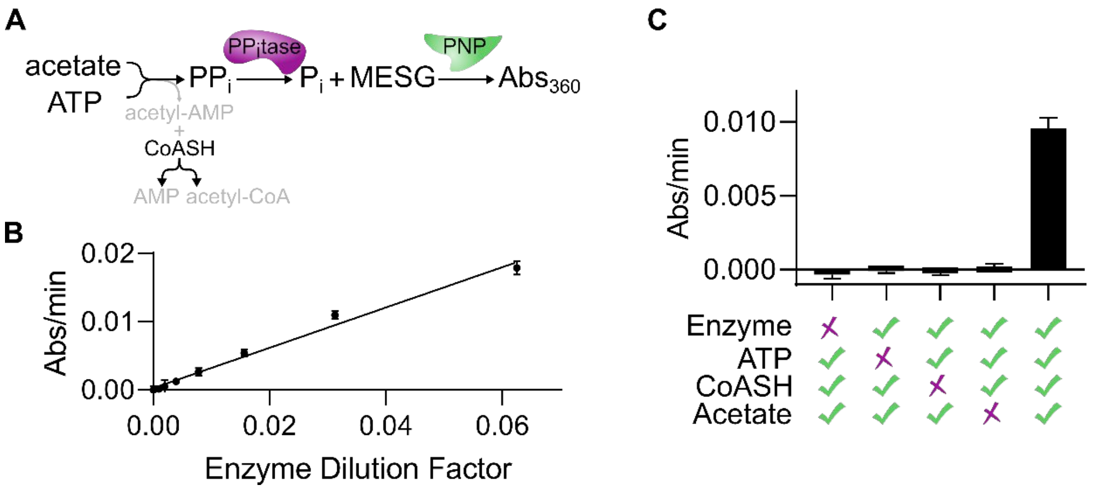

Supplemental Figure 2. ACS activity assay validation. (A) Schematic of ACS activity coupled to accessory enzyme detection of pyrophosphate (PPi) production. (B) Representative enzyme dilution curve to assure activity is measured within the linear range of enzyme content. (C) Detected activity is enzyme and substrate dependent.

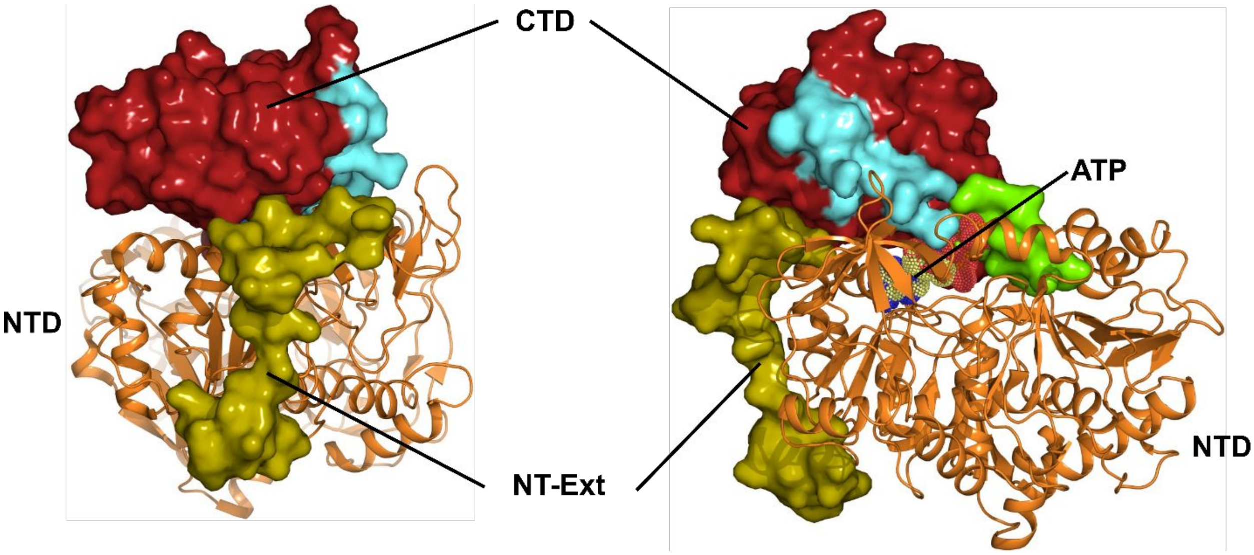

Supplementary Figure 3. N-terminal Extension (NT-Ext) from *Cryptococcus neoformans* Acs1. The ATP bound state (PDB 5K8F) is displayed with surfaces shown for NT-Ext (olive green), C-terminal domain (CTD, dark red), ATP binding loop (chartreuse) and hinge region (cyan). N-terminal domain (NTD) is shown in orange cartoon and ATP (yellow mesh).

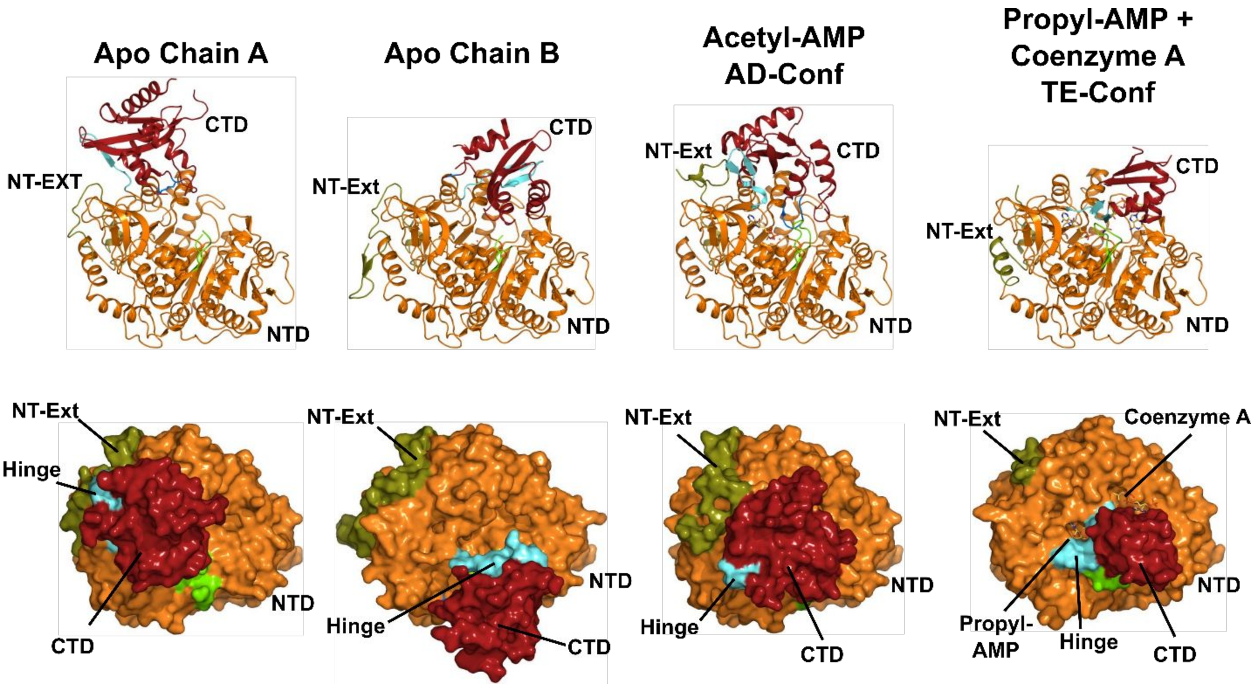

Supplemental Figure 4. Conformational orientations of the C-terminal Domain of *Cryptococcus neoformans* Acs1. (A-B) Apo (PDB 5PVP), (C) acetylating-conformation (AD-conf) bound to Acetyl-AMP (PDB 74LG) and (D) thioesterification conformation (TE-conf) bound to Propyl-AMP and Coenzyme A (PDB 5K85). N-terminal domain (NTD, orange), C-terminal domain (CTD, dark red), N-terminal extension (NT-Ext, olive green), hinge (cyan), and ATP binding loop (Chartreuse).

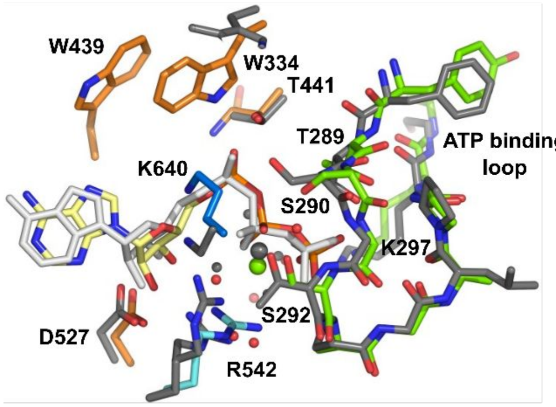

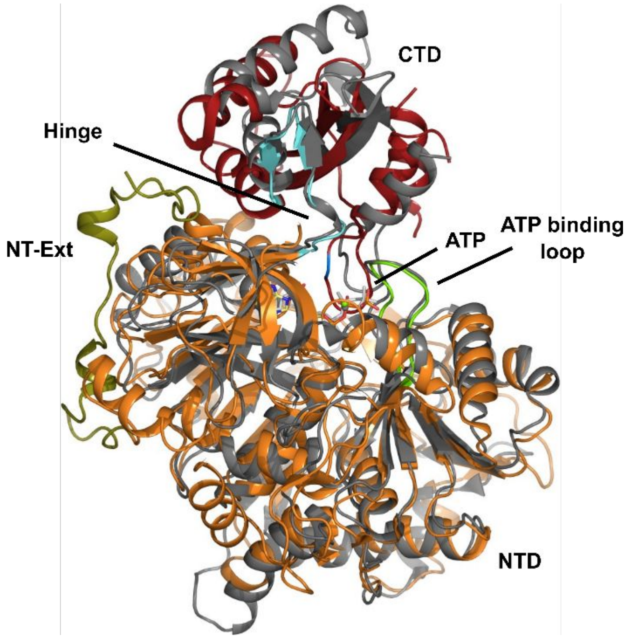

Supplemental Figure 5. Comparison of Human ACSM2A and *Cryptococcus neoformans* Acs1 bound to ATP and Magnesium. Human ACSM2A (PDB 3C5E) is shown in gray (cartoon) and light gray (compound). *Cryptococcus neoformans* Acs1 is shown in orange (N-terminal domain, NTD), dark red (C-terminal domain, CTD), olive green (N-terminal extension, NT-Ext), chartreuse (ATP binding loop), yellow (ATP), cyan (hinge), and blue (Active site Lys640). Right, overlay of N-terminal domain in PyMol shown in cartoon with (left) active site shown in stick. Residue numbering shown for *Cryptococcus neoformans* Acs1.

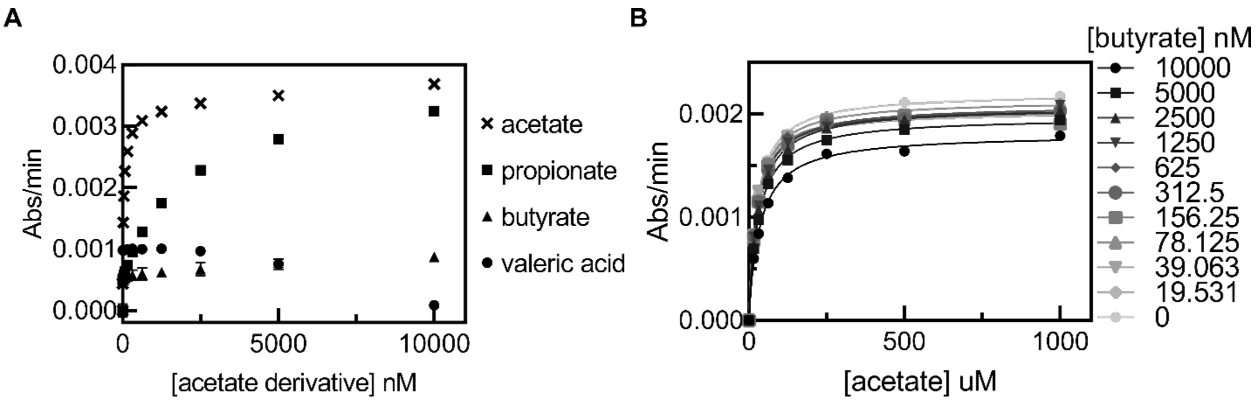

Supplemental Figure 6. Utilization of alternative acid substrates. (A) Activity of *Cn*Acs1 against alternative acid substrates. (B) The non-preferred acid substrate butyrate does not inhibit acetate utilization.

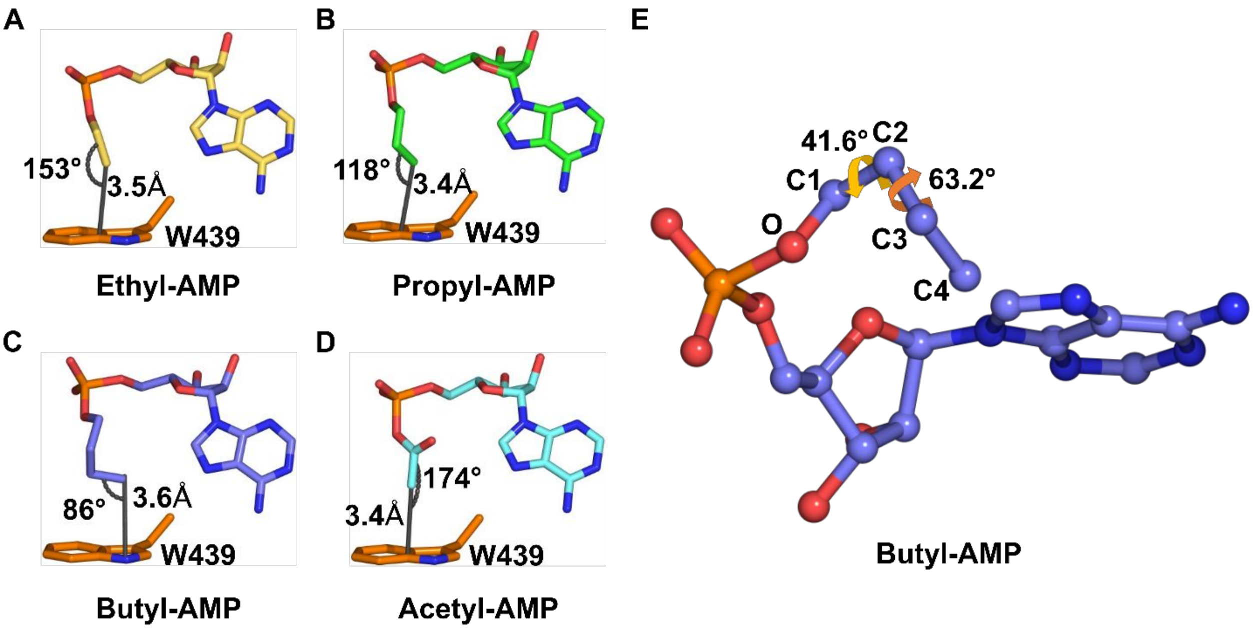

Supplemental Figure 7. Angle and distances from terminal methyl to Trp439 sidechain in *Cryptococcus neoformans* AD-conformation. (A) Ethyl-AMP (PDB 7NKO, Chain A), (B) Propyl-AMP (PDB 5IFI, Chain A), (C) Butyl-AMP (PDB 7KNP, Chain A), and (D) Acetyl AMP (PDB 74LG, Chain A). Angles were measured from the two carbon atoms nearest Trp439 (central atom always as the terminal carbon) and nearest atom on Trp439 side-chain (Ethyl, propyl, and acetyl = Cε2; Butyl = Nε1). Distances measured from terminal carbon atom and center of Trp439 ring (Ethyl, propyl and acetyl = center of both rings; Butyl = 5-membered ring only). (E) Dihedral angles measured for butyl AMP (PDB 7KNP) for O-C1-C2-C3 (yellow arrow) and C1-C2-C3-C4 (orange arrow).

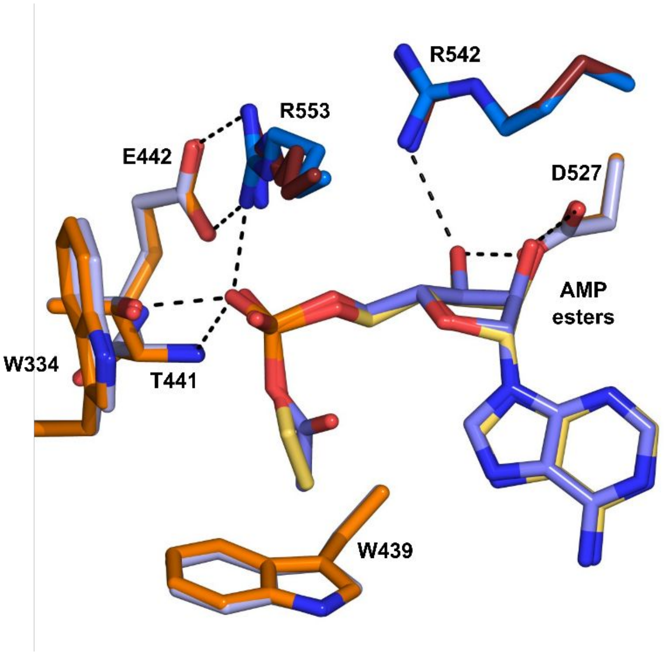

Supplemental Figure 8. Comparison of ethyl-AMP and acetyl-AMP bound to *Cryptococcus neoformans* Acs1. Overlay of ethyl-AMP (PDB 7KNO, compound – yellow, protein – orange) and acetyl-AMP (PDB 74LG, compound – blue, protein – light blue).

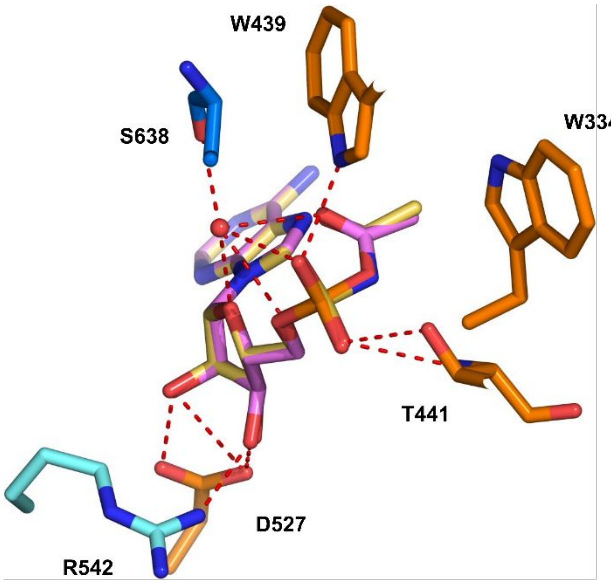

Cryptococcus neoformans
(AD-conf, Acetyl-AMS vs Acetyl-AMP

Supplemental Figure 9. Overlay of Acetyl-AMP and Acetyl-AMS crystal structures. Acetyl-AMP (PDB 74LG) and Acetyl-AMS (PDB 5U29) structures were overlaid in PyMol. Compounds are shown in stick representation with Acetyl-AMP in yellow and Acetyl-AMS in pink. Acetyl-AMS residues are shown surrounding the compound.

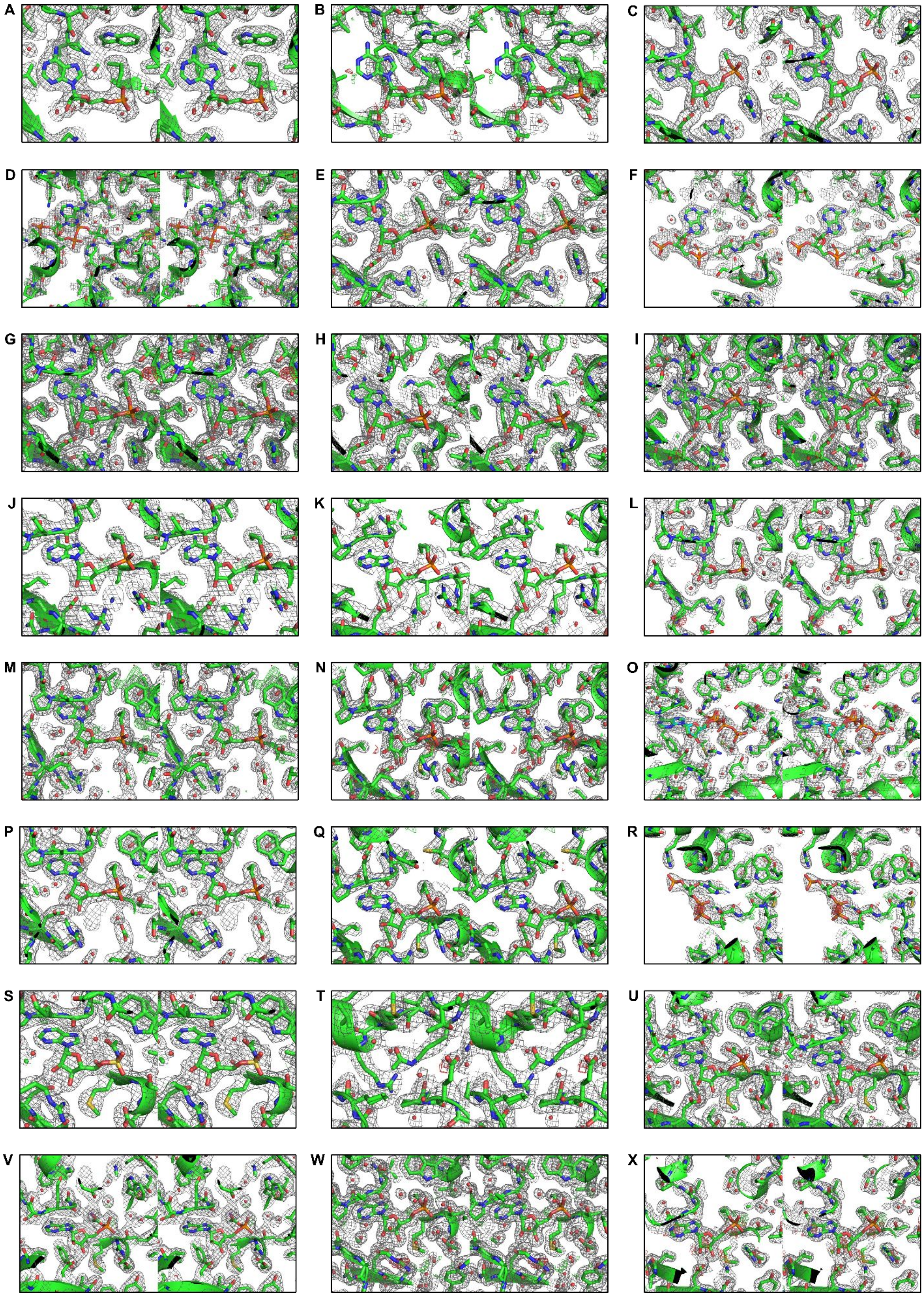
A
B
C
D
E
F
G
H
I
J
K
L
M
N
O
P
Q
R
S
T
U
V
W
X

Supplemental Figure 10. Stereo images of substrate binding pockets for fungal Acs1 crystal structures. *Cryptococcus neoformans* + butyl AMP (PDB 7KNP), (A) Chain A (AD conformation) and (B) Chain C (TE conformation). *Coccidioides immitis* + methyl AMP + coenzyme A (PDB 7L3Q), (C) Chain A, methyl AMP (TE conformation), and (D) Chain A, coenzyme A (TE conformation). *Coccidioides immitis* + ethyl AMP + coenzyme A (PDB 7KVY), (E) Chain A, ethyl AMP (TE conformation), and (F) Chain A, coenzyme A (TE conformation). *Coccidioides immitis* + propyl AMP (PDB 7KQ6), (G) Chain A (TE conformation). *Coccidioides immitis* + ethyl AMP (PDB 7KQZ), (H) Chain A (TE conformation). *Coccidioides immitis* + methyl AMP (PDB 7L3P), (I) Chain A (TE conformation). *Aspergillus fumigatus* + propyl AMP (PDB 7KDN), (J) Chain A (TE conformation). *Candida albicans* + propyl AMP (PDB 7KDS), (K) Chain A (TE conformation). *Coccidioides posadasii* + propyl AMP (PDB 7KCP), (L) Chain A (TE conformation). *Cryptococcus neoformans* + propyl AMP (PDB 5IFI), M) Chain A (AD conformation) and N) Chain C (TE conformation). *Cryptococcus neoformans* + ATP/acetyl AMP (PDB 5K8F), O) Chain A (AD conformation). *Cryptococcus neoformans* + propyl AMP + coenzyme A (PDB 5K85), P) Chain A (AD conformation) and Q) Chain C, propyl AMP (TE conformation), and R) Chain C, coenzyme A (TE conformation). *Cryptococcus neoformans* + acetyl AMS (PDB 5U29), S) Chain A (AD conformation). *Cryptococcus neoformans* + acetyl lysine (PDB 5VPV), T) Chain A. *Cryptococcus neoformans* + acetyl AMP (PDB 74LG), U) Chain A (AD conformation) and V) Chain C (TE conformation). *Cryptococcus neoformans* + ethyl AMP (PDB 7KNO), W) Chain A (AD conformation) and X) Chain C (TE conformation). 2Fo-Fc maps contoured at 1.0σ (grey), Fo-Fc maps contoured at +3.0/-3.0 (green/red). Stereo images generated in PyMol.

Supplemental Table 1. Crystallographic data and refinement statistics

Data name	*Cryptococcus neoformans*<br>Propyl-AMP<br>PDB 5IFI		*Cryptococcus neoformans*<br>ATP/Acetyl-AMP<br>PDB 5K8F		*Cryptococcus neoformans*<br>Propyl-AMP/CoA<br>PDB 5K85	
Data collection						
Wavelength (Å)	0.9787		0.9786		0.9786	
Space group	P 1 2_1 1		P 1		P 1 2_1 1	
Cell dimensions						
a, b, c (Å)	72.46 185.55 84.89		71.37 83.92 101.57		72.8 186.09 85.32	
α, β, γ (°)	90.0 93.7 90.0		110.1 105.2 87.6		90.0 93.8 90.0	
Resolution (Å)	50 - 1.95 (2.00 - 1.95)[a]		50 - 2.45 (2.51 - 2.45)		50 - 2.30 (2.36 - 2.30)	
No. of unique reflections	159,980 (11,779)		77,299 (5,646)		97,753 (7,375)	
R_{merge} (%)	5.8 (53.5)		7.6 (51.7)		7.2 (54.9)	
$I/\sigma(I)$	14.96 (2.47)		14.19 (2.56)		12.14 (2.01)	
Completeness (%)	98.7 (98.5)		98.5 (97.8)		97.5 (99.2)	
Redundancy	3.8 (3.9)		4.0 (4.0)		3.3 (3.2)	
CC½	99.8 (84.3)		99.8 (86.1)		99.7 (77.1)	
Refinement						
Resolution (Å)	50 – 1.95		50 – 2.45		50 – 2.30	
No. of unique reflections	159,842		77,260		97,663	
R_{work}/R_{free}[b]	0.185 (0.251)<br>0.213 (0.280)		0.188 (0.260)<br>0.224 (0.278)		0.179 (0.258)<br>0.217 (0.285)	
No. of atoms						
Protein	14,242		15,031		14,624	
Ligand	78		ATP<br>Acetyl AMP	93<br>78	Propyl AMP<br>CoA	78<br>48
Solvent	92		45		45	
Water	972		377		741	
B factors (Å²) (overall)						
Protein	43.4		53.03		48.74	
Ligand	37.7		ATP<br>Acetyl AMP	51.28<br>47.39	Propyl AMP<br>CoA	41.44<br>100.62
Solvent	63.4		48.51		60.81	
Water	40.4		38.93		42.51	
RMSD						
Bond lengths (Å)	0.007		0.002		0.007	
Bond angles (°)	1.15		0.48		1.12	
Ramachandran favored (%)	96.51		97.23		96.83	
Ramachandran allowed (%)	3.32		2.77		3.06	
Ramachandran outliers (%)	0.16		0		0.11	
Rotamer outliers (%)	0.28		1.46		1.02	

RMSD, root-mean-square deviation.
[a]Highest-resolution shell shown in parentheses.

[b]Rfree was calculated with 10% of the data or no greater than 2,000 reflections, whichever is lesser.

Supplemental Table 1. Crystallographic data and refinement statistics

Data name	*Cryptococcus neoformans* Acetyl-AMS PDB 5U29	*Cryptococcus neoformans* Apo PDB 5VPV	*Cryptococcus neoformans* Acetyl-AMP PDB 74LG
**Data collection**			
Wavelength (Å)	0.9786	0.9787	0.9787
Space group	P 1	P 4_1 2_1 2	P 1 2_1 1
Cell dimensions			
a, b, c (Å)	71.28 83.78 101.6	176.98 176.98 159.92	72.39 185.75 84.88
α, β, γ (°)	110.3 105.8 87.8	90.0 90.0 90.0	90.0 94.0 90.0
Resolution (Å)	50 – 2.50 (2.56 – 2.50)	50 - 2.60 (2.67 - 2.60)	50 - 2.20 (2.26 - 2.20)
No. of unique reflections	72,292 (5,310)	78,331 (5,723)	110,867 (8,124)
R_{merge} (%)	7.3 (58.8)	16.0 (52.1)	8.0 (62.5)
I/σ(I)	14.69 (2.64)	12.18 (5.02)	12.10 (2.25)
Completeness (%)	98.5 (97.8)	100.0 (100.0)	98.1 (97.3)
Redundancy	3.9 (4.0)	12.4 (12.7)	4.8 (4.8)
CC½	99.8 (78.9)	99.2 (95.2)	99.7 (84.6)
**Refinement**			
Resolution (Å)	50 – 2.50	50 – 2.60	50 – 2.20
No. of unique reflections	72,227	78,263	110,839
R_{work}/R_{free}^{b}	0.153 (0.236)<br>0.209 (0.309)	0.156 (0.182)<br>0.216 (0.281)	0.155 (0.239)<br>0.196 (0.313)
No. of atoms			
Protein	15,218	14,150	15,166
Ligand	78	N/A	78
Solvent	43	80	81
Water	402	685	1,032
B factors (Å²) (overall)			
Protein	52.02	35.91	45.54
Ligand	43.02	N/A	37.57
Solvent	75.68	69.15	53.89
Water	43.25	34.40	43.55
RMSD			
Bond lengths (Å)	0.007	0.007	0.007
Bond angles (°)	0.91	1.19	1.17
Ramachandran favored (%)	97.90	96.00	96.85
Ramachandran allowed (%)	2.10	3.78	3.04
Ramachandran outliers (%)	0	0.22	0.10
Rotamer outliers (%)	0.79	1.80	0.83

RMSD, root-mean-square deviation.

[a]Highest-resolution shell shown in parentheses.

[b]Rfree was calculated with 10% of the data or no greater than 2,000 reflections, whichever is lesser.

Supplemental Table 1. Crystallographic data and refinement statistics

Data name	*Cryptococcus neoformans* Ethyl-AMP PDB 7KNO	*Cryptococcus neoformans* Butyl-AMP PDB 7KNP	*Coccidioides immitis* Propyl-AMP PDB 7KQ6
Data collection			
Wavelength (Å)	0.9787	0.9787	0.9787
Space group	P 1 2_1 1	P 1 2_1 1	P 1 2_1 1
Cell dimensions			
a, b, c (Å)	72.69 184.7 84.84	72.25 184.66 85.13	106.98 116.08 107.33
α, β, γ (°)	90.0 93.9 90.0	90.0 93.8 90.0	90.0 119.8 90.0
Resolution (Å)	50 – 1.80 (1.85 – 1.80)	50 - 2.25 (2.31 - 2.25)	50 – 1.80 (1.85 – 1.80)
No. of unique reflections	205,272 (15,138)	103,490 (7,597)	201,793 (15,201)
R_{merge} (%)	5.2 (51.5)	9.0 (54.7)	4.8 (54.5)
I/σ(I)	15.12 (2.31)	10.14 (2.60)	15.73 (2.10)
Completeness (%)	99.8 (99.9)	98.4 (97.9)	95.8 (97.9)
Redundancy	3.8 (3.7)	3.9 (3.9)	3.1 (3.0)
CC½	99.9 (84.9)	99.5 (81.0)	99.9 (74.4)
Refinement			
Resolution (Å)	50 – 1.80	50 – 2.25	50 – 1.80
No. of unique reflections	205,190	103,450	201,774
R_{work}/R_{free}[b]	0.164 (0.261)<br>0.190 (0.285)	0.176 (0.235)<br>0.213 (0.267)	0.162 (0.240)<br>0.185 (0.288)
No. of atoms			
Protein	14,987	13,988	14,057
Ligand	75	81	78
Solvent	57	58	128
Water	1,761	822	1,556
B factors (Å²) (overall)			
Protein	37.82	53.84	31.65
Ligand	28.84	51.53	24.79
Solvent	46.77	52.46	34.87
Water	39.26	44.22	36.39
RMSD			
Bond lengths (Å)	0.008	0.007	0.007
Bond angles (°)	0.90	0.83	0.80
Ramachandran favored (%)	96.86	96.18	95.88
Ramachandran allowed (%)	2.98	3.71	4.12
Ramachandran outliers (%)	0.16	0.11	0
Rotamer outliers (%)	0.13	0.85	0.55

RMSD, root-mean-square deviation.

[a]Highest-resolution shell shown in parentheses.

[b]Rfree was calculated with 10% of the data or no greater than 2,000 reflections, whichever is lesser.

Supplemental Table 1. Crystallographic data and refinement statistics			
Data name	*Coccidioides immitis* Ethyl-AMP PDB 7KQZ	*Coccidioides immitis* Methyl-AMP PDB 7L3P	*Coccidioides immitis* Methyl-AMP/CoA PDB 7L3Q
Data collection			
Wavelength (Å)	0.9787	0.9787	0.9787
Space group	P 6_3	P 6_3	P 1 2_1 1
Cell dimensions			
a, b, c (Å)	107.17 107.17 116.17	107.57 107.57 116.48	106.44 116.27 106.82
α, β, γ (°)	90.0 90.0 120.0	90.0 90.0 120.0	90.0 119.7 90.0
Resolution (Å)	50 – 2.15 (2.21 – 2.15)	50 - 2.10 (2.15 - 2.10)	50 – 2.15 (2.21 – 2.15)
No. of unique reflections	41,174 (3,016)	44,619 (3,324)	115,434 (8,805)
R_{merge} (%)	6.7 (55.7)	5.9 (62.8)	5.3 (56.5)
I/σ(I)	21.83 (4.24)	22.95 (3.72)	13.74 (2.24)
Completeness (%)	100.0 (100.0)	99.9 (88.6)	93.9 (97.4)
Redundancy	9.0 (9.0)	8.9 (8.9)	3.1 (3.0)
CC½	99.9 (90.3)	99.9 (88.6)	99.8 (70.7)
Refinement			
Resolution (Å)	50 – 2.15	50 – 2.10	50 – 2.15
No. of unique reflections	41,142	44,615	115,426
R_{work}/R_{free}[b]	0.154 (0.195)<br>0.192 (0.238)	0.160 (0.221)<br>0.195 (0.248)	0.187 (0.277)<br>0.233 (0.318)
No. of atoms			
Protein	4,707	4,636	14,447
Ligand	25	24	Methyl-AMP 72<br>Coenzyme A 144
Solvent	20	37	64
Water	323	298	541
B factors (Å²) (overall)			
Protein	50.19	51.21	50.77
Ligand	39.78	45.79	Methyl AMP 43.17<br>Coenzyme A 66.31
Solvent	45.14	51.31	41.51
Water	44.35	46.68	40.76
RMSD			
Bond lengths (Å)	0.007	0.007	0.008
Bond angles (°)	1.16	0.80	1.19
Ramachandran favored (%)	96.26	96.27	95.83
Ramachandran allowed (%)	3.57	3.56	4.17
Ramachandran outliers (%)	0.17	0.17	0
Rotamer outliers (%)	0.64	0.85	0.69

RMSD, root-mean-square deviation.

[a]Highest-resolution shell shown in parentheses.

[b]Rfree was calculated with 10% of the data or no greater than 2,000 reflections, whichever is lesser.

Supplemental Table 1. Crystallographic data and refinement statistics

Data name	*Coccidioides immitis* Ethyl-AMP/CoA PDB 7KVY	*Aspergillus fumigatus* Propyl-AMP PDB 7KDN	*Candida albicans* Propyl-AMP PDB 7KDS
**Data collection**			
Wavelength (Å)	0.9787	0.9787	0.9795
Space group	$P\ 6_3$	P 1	$P\ 4_1\ 3\ 2$
Cell dimensions			
a, b, c (Å)	106.94 106.94 115.96	103.79 104.21 125.65	166.92 166.92 166.92
α, β, γ (°)	90.0 90.0 120.0	68.154 66.546 62.224	90.0 90.0 90.0
Resolution (Å)	50 – 1.90 (1.95 – 1.90)	50 - 2.80 (2.87 - 2.80)	50 – 2.90 (2.98 – 2.90)
No. of unique reflections	59,143 (4,345)	100,485 (7,598)	18,239 (1,305)
R_{merge} (%)	5.3 (86.0)	12.0 (93.7)	8.1 (261.7)
I/σ(I)	18.29 (2.09)	10.14 (1.57)	34.52 (1.55)
Completeness (%)	100.0 (100.0)	97.9 (97.5)	99.9 (100.0)
Redundancy	5.7 (5.7)	3.9 (4.0)	38.5 (35.8)
CC½	99.9 (68.8)	99.6 (70.3)	100.0 (63.4)
**Refinement**			
Resolution (Å)	50 – 1.90	50 – 2.80	50 – 2.90
No. of unique reflections	59,133	100,431	18,188
R_{work}/R_{free}[b]	0.163 (0.256)<br>0.193 (0.297)	0.216 (0.355)<br>0.246 (0.476)	0.232 (0.350)<br>0.267 (0.359)
No. of atoms			
Protein	4,917	26,464	4,801
Ligand	Ethyl AMP 25<br>Coenzyme A 48	156	26
Solvent	36	N/A	1
Water	351	43	4
B factors (Å²) (overall)			
Protein	45.71	65.79	118.46
Ligand	Ethyl AMP 35.93<br>Coenzyme A 56.25	64.74	109.02
Solvent	45.71	N/A	179.85
Water	44.40	52.71	99.77
RMSD			
Bond lengths (Å)	0.008	0.004	0.004
Bond angles (°)	1.15	0.96	0.94
Ramachandran favored (%)	96.17	96.94	97.22
Ramachandran allowed (%)	3.83	3.06	2.78
Ramachandran outliers (%)	0	0	0
Rotamer outliers (%)	1.00	1.39	1.08

RMSD, root-mean-square deviation.

[a]Highest-resolution shell shown in parentheses.

[b]Rfree was calculated with 10% of the data or no greater than 2,000 reflections, whichever is lesser.

Supplemental Table 1. Crystallographic data and refinement statistics	
Data name	*Coccidioides posadasii* Propyl-AMP PDB 7KCP
Data collection	
Wavelength (Å)	0.9787
Space group	P 6_3
Cell dimensions	
a, b, c (Å)	107.17 107.17 116.17
α, β, γ (°)	90.0 90.0 120.0
Resolution (Å)	50 – 2.15 (2.21 – 2.15)
No. of unique reflections	41,158 (3,025)
R_{merge} (%)	8.1 (58.1)
I/σ(I)	20.04 (4.20)
Completeness (%)	100.0 (100.0)
Redundancy	9.0 (9.0)
CC½	99.9 (90.5)
Refinement	
Resolution (Å)	50 – 2.15
No. of unique reflections	41,145
R_{work}/R_{free}[b]	0.154 (0.195)<br>0.192 (0.238)
No. of atoms	
Protein	4,604
Ligand	26
Solvent	45
Water	371
B factors (Å^2) (overall)	
Protein	42.88
Ligand	34.32
Solvent	44.80
Water	41.07
RMSD	
Bond lengths (Å)	0.007
Bond angles (°)	1.16
Ramachandran favored (%)	96.26
Ramachandran allowed (%)	3.57
Ramachandran outliers (%)	0.17
Rotamer outliers (%)	0.64

RMSD, root-mean-square deviation.

[a]Highest-resolution shell shown in parentheses.

[b]Rfree was calculated with 10% of the data or no greater than 2,000 reflections, whichever is lesser.

Supplementary Table 1.

Species	Species						
	Cn	*Af*	*Ca*	*Ci*	*Cp*	*Sc*	*Se*
C. neoformans (*Cn*)	-	63.13 %	58.95 %	59.97 %	59.65 %	56.50 %	51.03 %
A. fumigatus (*Af*)	0.61 Å	-	63.29 %	82.26 %	82.71 %	62.99 %	51.10 %
C. albicans (*Ca*)	0.56 Å	0.53 Å	-	61.24 %	61.24 %	59.97 %	49.84 %
C. immitis (*Ci*)	0.55 Å	0.30 Å	0.51 Å	-	99.13 %	63.41 %	50.94 %
C. posadasii (*Cp*)	0.55 Å	0.30 Å	0.51 Å	0.11 Å	-	63.26 %	51.27 %
S. cerevisiae (*Sc*)	0.60 Å	0.55 Å	0.56 Å	0.56 Å	0.55 Å	-	46.55 %
S. enterica (*Se*)	0.66 Å	0.71 Å	0.76 Å	0.66 Å	0.61 Å	0.72 Å	-

Supplementary Table 2. Structural and Sequence comparison of Acs1. Sequence alignment performed by NCBI blast pairwise alignment (Ref) with percent identity shown in the upper right section of the table. Alignment performed by PyMol (Ref) of Cα atoms in the N-terminal extension and N-terminal domain of pairwise species expressed as RMSD (Å) in the bottom left section of the table.

```
Crne J9VFT1   1 M------------GKTEV-APGVHHVHPLPDSVPESEDLFAP-------PPRMQGKEGRP
Coim J3KJM0   1 MS----------ETPAEP-KLPVVVEAHQVDTFDVPGVFYEN----------------HP
Copo C5PGB4   1 MS----------ETPAEP-KPPVVVEAHQVDTFDVPGVFYEN----------------HP
Asfu Q4WQ02   1 MS----------DGDVKPAKPAVVLEANDVDTFHVPKAFYEK----------------HP
Caal Q8NJN3   1 M-------------PTEQ-THNVVHEANGVKLRETPKEFFER----------------QP
Sace Q01574   1 MSPSAVQSSKLEEQSSEIDKLKAKMSQSAATAQQKKEHEYEHLTSVKIVPQRPISDRLQP

Crne J9VFT1  40 ------KPHIGPNYESYVKEWAKTVGPNSDEWWAAKARETLDWYDDFKTVRAGGFEHG--
Coim J3KJM0  34 H-----EPHLS-GMNEYNQLYQQSI-NDPDTFWARMARDLITFEKDFDKTHIGTLEGG--
Copo C5PGB4  34 H-----EPHLS-GMNEYNQLYQQSI-NDPDTFWARMARDLITFEKDFDKTHIGTLEGG--
Asfu Q4WQ02  35 S-----KTHLK-DLDEYKKLYDESI-RSPDTFWARMARELLTFDKDFQTTHIGSLENG--
Caal Q8NJN3  31 N-----KGHIH-DVNQYKQMYEQSI-KDPQGFFGPLAKELLSWDHDFHTVKSGTLKNG--
Sace Q01574  61 AIATHYSPHLD-GLQDYQRLHKESI-EDPAKFFGSKATQFLNWSKPFDKVFIPDPKTGRP
                              α1                 α2                 β1

                                                                A1
Crne J9VFT1  93 ---DVQWFPEGTLNAAYNCLDRHYYKNPKKTAIIYEADEPSESREVSYEELMQETCRVAN
Coim J3KJM0  85 ---DNAWFVGGRLNASFNCVDRHAMRDPNKVAIIYEADEPGHGRSITYAELLKEVSRLAW
Copo C5PGB4  85 ---DNAWFVGGRLNASFNCVDRHAMRDPNKVAIIYEADEPGHGRSITYAELLKEVSRLAW
Asfu Q4WQ02  86 ---DNAWFVEGRLNASFNCVDRHAIKNPNKVAIIYEADEPNEGRIITYGELLREVSRVAW
Caal Q8NJN3  82 ---DAAWFLGGELNASYNCVDRHAFANPDKPALICEADDEKDSHILTYGDLLREVSKVAG
Sace Q01574 119 SFQNNAWFLNGQLNACYNCVDRHALKTPNKKAIIFEGDEPGQGYSITYKELLEEVCQVAQ
                  β2            α3             β3     α4   β4      α5

                                          A2
1
2                                                  o o
Crne J9VFT1 150 VLK-SYGVKKGDAVSIYLPMTWQAAAAFLACARIGAIHSAVFAGFSAESLRDRVNDCECK
Coim J3KJM0 142 VMK-SQGVRKGDTVAIYLPMIPEAIFALLACARIGAIHSVVFAGFSSDSLRDRTLDARSK
Copo C5PGB4 142 VMK-SQGVRRGDTVAIYLPMIPEAIFALLACARIGAIHSVVFAGFSSDSLRDRTLDARSK
Asfu Q4WQ02 143 VLK-QRGVKKGDTVAIYLPMIPEAVVAFLACARIGAIHSVVFAGFSSDSLRDRVLDAGSK
Caal Q8NJN3 139 VLQ-SWGIKKGDTVAVYLPMNAQAIIAMLAIARLGAAHSVIFAGFSAGSIKDRVNDASCK
Sace Q01574 179 VLTYSMGVRKGDTVAVYMPMVPEAIITLLAISRIGAIHSVVFAGFSSNSLRDRINDGDSK
                 α5      β5            α6          β6          α7

1
2                     o
Crne J9VFT1 209 VLITTDEGRRGGKTIATKQIVDAALQQCPLVENVLVLRRT-GNKVPMTEGRDKWWDEECA
Coim J3KJM0 201 FIITTDEGKRGGKVIGTKKIVDEALKQCPDVTNCLVFKRT-GADVPWTKGRDLWWHEEVD
Copo C5PGB4 201 FIITTDEGKRGGKVIGTKKIVDEALKQCPDVTSCLVFKRT-GADVPWTKGRDLWWHEEVE
Asfu Q4WQ02 202 VVITTDEGKRGGKVIGTKRIVDEALKQCPDVTSVLVYKRT-GAEVPWTNGRDIWWHEEVE
Caal Q8NJN3 198 ALITCDEGKRGGRTTNIKKLCDEALVDCPTVEKVLVYKRTNNPEIHLTEGRDYYWDVETA
Sace Q01574 239 VVITTDESNRGGKVIETKRIVDDALRETPGVRHVLVYRKTNNPSVAFHAPRDLDWATEKK
                 β7   β8   β9     α8            β10            β11 α9
```

```
                                     A3
1                    •••••   •
2
Crne J9VFT1 268 KMPAYCPCERMASEDPLFILYTSGSTGKPKGVVHSTAGYLLGTALTLKYVFDAHPDDRFA
Coim J3KJM0 260 KYPNYLPAESMDSEDPLFLLYTSGSTGKPKGVMHTTAGYLVGAAATGKYVFDIHPADRFF
Copo C5PGB4 260 KYPNYLPAESMDSEDPLFLLYTSGSTGKPKGVMHTTAGYLVGAAATGKYVFDIHPADRFF
Asfu Q4WQ02 261 KYPCYVAPESMSSEDPLFLLYTSGSTGKPKGVMHTTAGYLLGAAMTGKYVFDIHDDDRFF
Caal Q8NJN3 258 KFPGYLPPVSVNSEDPLFLLYTSGSTGTPKGVVHSTAGYLLGAALSTKYIFDIHPEDILF
Sace Q01574 299 KYKTYYPCTPVDSEDPLFLLYTSGSTGAPKGVQHSTAGYLLGALLTMRYTFDTHQEDVFF
```

α9 β12 β13 β14 α10 β15

```
                      A4
1                     Ø××
2                 oo  o‡»                       o                        oo
Crne J9VFT1 328 CMADIGWITGHSYIIYGPLANGITTAVFESTPVYPTPSRYWDFVDKWKATQLYTAPTAIR
Coim J3KJM0 320 CGGDVGWITGHTYVVYAPLLLGCTTVVFESTPAYPNFSRYWDVIEKHKVTQFYVAPTALR
Copo C5PGB4 320 CGGDVGWITGHTYVVYAPLLLGCTTVVFESTPAYPNFSRYWDVIEKHKVTQFYVAPTALR
Asfu Q4WQ02 321 CGGDVGWITGHTYVVYAPLLLGCSTVVFESTPAYPNFSRYWDVIEKHKVTQFYVAPTALR
Caal Q8NJN3 318 TAGDVGWITGHTYALYGPLLLGVPTIIFEGTPAYPDYGRFWQIVEKHKATHFYVAPTALR
Sace Q01574 359 TAGDIGWITGHTYVVYGPLLYGCATLVFEGTPAYPNYSRYWDIIDEHKVTQFYVAPTALR
```

β15 α11 β16 α12 β17 α13

```
                                                                A5
1                                      ×ØØØ                     ØØØØØØ
2                                      ‡‡‡‡                     ‡‡‡‡‡‡
Crne J9VFT1 388 LLRRMGEDHVKNHDLSSLRVLGSVGEPINPEAWHWYNDFAGKNQCAIVDTYWMTETGSIS
Coim J3KJM0 380 LLKRAGDHHI-NHEMKDLRILGSVGEPIAAEVWKWYHEVVGKRQAHIVDTYWQTETGSHV
Copo C5PGB4 380 LLKRAGDHHI-NHEMKDLRILGSVGEPIAAEVWKWYHEVVGKRQAHIVDTYWQTETGSHV
Asfu Q4WQ02 381 LLKRAGDEHI-HHKMEHLRILGSVGEPIAAEVWKWYFEKVGKEEAHICDTYWQTETGSHV
Caal Q8NJN3 378 LLRKAGEQEIAKYDLSSLRTLGSVGEPISPDIWEWYNEFVGKNQCHISDTYWQTESGSHL
Sace Q01574 419 LLKRAGDSYIENHSLKSLRCLGSVGEPIAAEVWEWYSEKIGKNEIPIVDTYWQTESGSHL
```

α13 α14 β18 α15 β19 α16

```
                                                                A6
Crne J9VFT1 448 IAPL-PGAISTKPGSATFPFFGMDVDIIDPQTGQVLEGNDVEGVLVARRPWPSIARTVYR
Coim J3KJM0 439 ITPL-GGITPTKPGSASLPFFGIDPVILDPVTGAEIPGNDVEGILAFRKPWPSMARTVWG
Copo C5PGB4 439 ITPL-GGITPTKPGSASLPFFGIDPVILDPVTGAEIPGNDVEGILAFRKPWPSMARTVWG
Asfu Q4WQ02 440 ISPL-GGITSTKPGSASLPFFGIEPAIIDPVSGEEISGNDVEGVLAFKQPWPSMARTVWG
Caal Q8NJN3 438 IAPL-AGVVPNKPGSASYPFFGIDAALIDPVTGVEIEGNDAEGVLAIKDHWPSMARTVYK
Sace Q01574 479 VTPLAGGVTPMKPGSASFPFFGIDAVVLDPNTGEELNTSHAEGVLAVKAAWPSFARTIWK
```

β20 β21 β22

```
                                  A7                   A8
1                                   Ø           Ø  Ø
2                                   ‡           ‡  ‡     ‡ ooo‡
Crne J9VFT1 507 DHKRYLETYMKPYPGYFFFGDGAARDYDGYMWIKGRVDDVINVSGHRLSTAEVESALILH
Coim J3KJM0 498 DHKRYMDTYLNVYKGFYFTGDGAGRDHEGYYWIRGRVDDVVNVSGHRLSTAEIEAALIEH
Copo C5PGB4 498 DHKRYMDTYLNVYKGFYFTGDGAGRDHEGYYWIRGRVDDVVNVSGHRLSTAEIEAALIEH
Asfu Q4WQ02 499 AHKRYMDTYLNVYKGYYFTGDGAGRDHDGYYWIRGRVDDVVNVSGHRLSTAEIEAALIEH
Caal Q8NJN3 497 NHTKYMDTYMNPYPGYYFTGDGAARDHDGYYWIRGRVDDVVNVSGHRLSTAEIEAALIED
Sace Q01574 539 NHDRYLDTYLNPYPGYYFTGDGAAKDKDGYIWILGRVDDVVNVSGHRLSTAEIEAAIIED
                   α17         β23          β24   α18 β25 β26    α19

                                                                       A9
1
2                                                                 o    oo
Crne J9VFT1 567 KGVAETAVVGCADDLTGQAVYAFVTMKPEFDLK---ATKEADLSKELAIQVRKVIGPFAA
Coim J3KJM0 558 HCVAEAAVVGVPDPLTGQAVHAFVALKSGN-------DNREQLQKELIMQVRKSIGPFAA
Copo C5PGB4 558 HCVAEAAVVGVPDPLTGQAVHAFVALKSGN-------DNREQLRKELIMQVRKSIGPFAA
Asfu Q4WQ02 559 HQVAEAAVVGIADELTGQAVNAFVSLKDGN-------ETTEQVRKDLVMQVRKSIGPFAA
Caal Q8NJN3 557 KKVSEAAVVGIHDDITGQAVIAYVALKEGNS-----DEDSEGLRKELVLQVRKTIGPFAA
Sace Q01574 599 PIVAECAVVGFNDDLTGQAVAAFVVLKNKSSWSTATDDELQDIKKHLVFTVRKDIGPFAA
                     β27           β28                         α20

                            A10
1                               •
2
Crne J9VFT1 624 PKKIYLVSDLPKTRSGKIMRRVLRKIVAGEGDQLGDLSSIADPQIVEEVKQKVT------
Coim J3KJM0 611 PKVVFVIDDLPKTRSGKIMRRILRKILSGEEDSLGDISTLSDPSVVNKIIDTFHEWKKAM
Copo C5PGB4 611 PKVVFVIDDLPKTRSGKIMRRILRKILSGEEDSLGDISTLSDPSVVNKIIDTFHEWKRAM
Asfu Q4WQ02 612 PKAVFVVEDLPKTRSGKIMRRILRKILSGEEDSLGDTSTLSDPSVVNKIIETVH------
Caal Q8NJN3 612 PKSVIIVQDLPKTRSGKIMRRILRKVSSNEADQLGDISTLSNPQSVEGIISAFG------
Sace Q01574 659 PKLIILVDDLPKTRSGKIMRRILRKILAGESDQLGDVSTLSNPGIVRHLIDSVK------
                  β29                α21                       α22

Crne J9VFT1 678 -------GS-----------A 680
Coim J3KJM0 671 AAAAAAAAAVSATAPPNSTTG 691
Copo C5PGB4 671 AAAAAAAAAVSATAPPNSTTG 691
Asfu Q4WQ02 666 ----AARA------------K 670
Caal Q8NJN3 666 ----AQFGK-----------K 671
Sace Q01574 713 --------------------L 713
```

Supplemental Table 3. Alignment of fungal Acs1. Multiple sequence alignment by MAFFT (Ref) with *Cryptococcus neoformans* (Uniprot ID J9VFT1), *Aspergillus fumigatus* (Uniprot ID Q4WQ02), *Candida albicans* (Uniprot ID Q8NJN3), *Coccidioides immitis* (Uniprot ID J3KJMO), *Coccidioides posadasii* (Uniprot ID C5PGB4), and *Saccharomyces cerevisiae* (Uniprot ID Q01574). Secondary structure shown below the alignment as red rectangles (alpha helices) and

yellow arrows (beta strands). Identical residues are in red font with gray background and chemically similar compounds are in blue font with gray background. Boxes over the alignment indicate conserved Acs1 enzyme family motifs labeled A1-A10. Symbols above the alignment indicate ligand or substrate interacting residues with “1” for the Acetylation reaction conformation (•) ATP only, (×) Acetyl-AMP only and, (Ø) both ligands; “2” for the thioesterification reaction conformation. (o) Coenzyme A only, (‡) Acetyl-AMP only, (») both.

**Species**	**Compound IC_{50} (μM)**	
	ethyl-AMP	butyl-AMP
C. neoformans	2.10	> 50
A. fumigatus	0.06	> 50
C. albicans	0.29	> 50
C. immitis	0.06	> 50
C. posadasii	0.07	> 50

Supplemental Table 4. Potency of ethyl-AMP and butyl-AMP across fungal Acs1 recombinant proteins

Synthetic Procedures for the Preparation of AMP alkyl esters, AMS and AcAMS.

All chemicals were purchased from Sigma Aldrich and were used without any further purification. 5'-O-Sulfamoyl-2', 3'-O-isopropylideneadenosine, N-acetoxysuccinimide, AMS and Ac-AMS were prepared by the method of Qiao (1). All tested compounds have purity of >95% as determined by HPLC analysis (UV detection @254 nM). Purification of compounds was done on a Biotage® Isolera using a Biotage® SNAP cartridge KP-Sil 50g. Purity of compounds was determined using an Agilent HPLC utilizing a C-18 column (Waters Nova-Pak; 3.9 x 100 mm) with the following method: Solvent A = H_2O (0.1% TFA), Solvent B = MeCN; 0 to 20 min, (10 to 90% B), 20 to 25 min (90 to 10% B); detection was set at two wavelengths (254 and 280 nm). ^1H and ^{13}C NMR were recorded on a BrukerAvance III 500 outfitted with a 5mm BBFO Z-gradient probe. Chemical shifts δ are in ppm, and spectra are referenced using the residual solvent peak. The following abbreviations are used: singlet (s), doublet (d), triplet (t), quartet (q), double doublet (dd), quintet (quin), multiplet (m), broad signal (br s). High-resolution mass spectra (HRMS) were obtained on a Bruker Maxis Plus Quadrupole Time-of-Flight (QTOF).

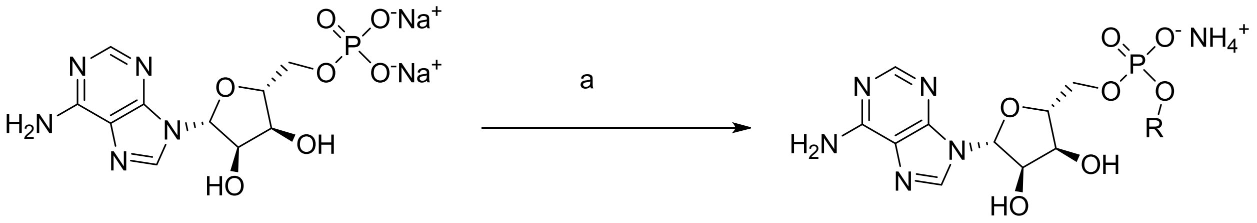

1a (R=CH_3)
1b (R=CH_2CH_3)
1c (R=$CH_2CH_2CH_3$)
1d (R=$CH_2CH_2CH_2CH_3$)

Reagent and reaction conditions: (a) Corresponding alcohol, EDC (5.0 eq), rt, 24 h.

General procedure for preparation of adenosine 5'-alkyl phosphate (1a-1d)

To a stirred solution of adenosine monophosphate disodium salt (1.00 g, 2.56 mmol) in 150 mL of corresponding alcohol was added EDC (2.45 g, 12.78 mmol). The reaction was stirred for 24

hr. followed by removal of the solvent under reduced under pressure. The resulting solid was purified by column chromatography on a Biotage Isolera using a Biotage SNAP cartridge KP-Sil 50g using a linear gradient of 80:20 to 70:30 (CH_2Cl_2/MeOH with 4% NH_4OH) to afford the corresponding compounds **1a-1d** as white solids.

Adenosine 5'-methyl phosphate (Methyl-AMP) (1a)

Yield; 63%,^{1}H NMR (500 MHz, DMSO-*d6*) δ 8.46 (s, 1H), 8.15 (s, 1H), 7.31 (s, 2H), 5.92 (d, *J*= 6.0 Hz, 1H), 5.54 (s, 1H), 5.43(s, 1H), 4.60 (t, J = 6.9, 1H), 4.20 (t, *J* = 4.0Hz), 4.02 (q, *J* = 4.0Hz), 3.84 (m, 1H), 3.80 (m, 1H), 3.31 (d, *J* = 10.5Hz, 3H), ^{13}C NMR (125 MHz, DMSO-*d6*), 156.4, 153.1, 150.1, 119.3, 87.4, 84.4, 74.4, 71.3, 65.0, 52.1, HRMS for $C_{11}H_{16}N_5O_7P$ $[M+H]^+$ calculated; 362.0866, found; 362.0855

Adenosine 5'-ethyl phosphate (Ethyl-AMP) (1b)

Yield; 46%,^{1}H NMR (500 MHz, DMSO-*d6*) δ 8.46 (s, 1H), 8.15 (s, 1H), 7.30 (s, 2H), 5.92 (d, *J*= 6.0 Hz, 1H), 5.56 (s, 1H), 5.55(s, 1H), 4.59 (t, J = 6.9, 1H), 4.19 (t, *J* = 4.0Hz), 4.03 (q, *J* = 4.0Hz), 3.87 (m, 1H), 3.79 (m, 1H), 3.68 (m, 2H), 1.08 (t, J= 7.0 Hz, 3H), ^{13}C NMR (125 MHz, DMSO-*d6*), 156.4, 153.1, 150.1, 139.8, 119.3, 87.4, 84.3, 74.3, 71.2, 64.9, 60.3, 17.0, HRMS for $C_{12}H_{18}N_5O_7P$ $[M+H]^+$ calculated; 376.1023, found; 376.1033

Adenosine 5'- propyl phosphate (Propyl-AMP) (1c)

Yield; 43%,^{1}H NMR (500 MHz, DMSO-*d6*) δ 8.44 (s, 1H), 8.15 (s, 1H), 7.30 (s, 2H), 5.91 (d, *J*= 6.0 Hz, 1H), 5.57 (s, 1H), 5.56 (s, 1H), 4.59 (t, J = 6.9, 1H), 4.19 (t, *J* = 4.0Hz), 4.02 (q, *J* = 4.0Hz), 3.87 (m, 1H), 3.78 (m, 1H), 3.58 (q, *J* = 6.7 Hz, 2H), 1.45 (m, 2H), 0.79 (t, J= 7.5 Hz, 3H), ^{13}C NMR (125 MHz, DMSO-*d6*), 156.4, 153.1, 150.0, 139.8, 119.3, 87.5, 84.2, 74.2, 71.2, 66.4, 65.0, 24.0, 10.8, HRMS for $C_{13}H_{20}N_5O_7P$ $[M+H]^+$ calculated; 390.1179 , found; 390.1192

Adenosine 5'- butyl phosphate (Butyl-AMP) (1d)

Yield; 35%,^{1}H NMR (500 MHz, DMSO-*d6*) δ 8.44 (s, 1H), 8.15 (s, 1H), 7.29 (s, 2H), 5.91 (d, *J*= 6.0 Hz, 1H), 5.52 (s, 1H), 5.42(s, 1H), 4.58 (t, J = 6.9, 1H), 4.19 (t, *J* = 4.0 Hz), 4.02 (q, *J* = 4.0 Hz), 3.86 (m, 1H), 3.78 (m, 1H), 3.61 (q, *J* = 6.7 Hz, 2H), 1.43 (m, 2H), 1.24 (m, 2H), 0.81 (t, J= 7.5 Hz, 3H), ^{13}C NMR (125 MHz, DMSO-*d6*), 156.4, 153.1, 150.0, 139.9, 119.3, 87.5, 84.1, 74.2, 71.2, 65.1, 64.5, 32.9, 19.0, 14.1, HRMS for $C_{14}H_{22}N_5O_7P$ $[M+H]^+$ calculated; 404.1336, found; 404.1342

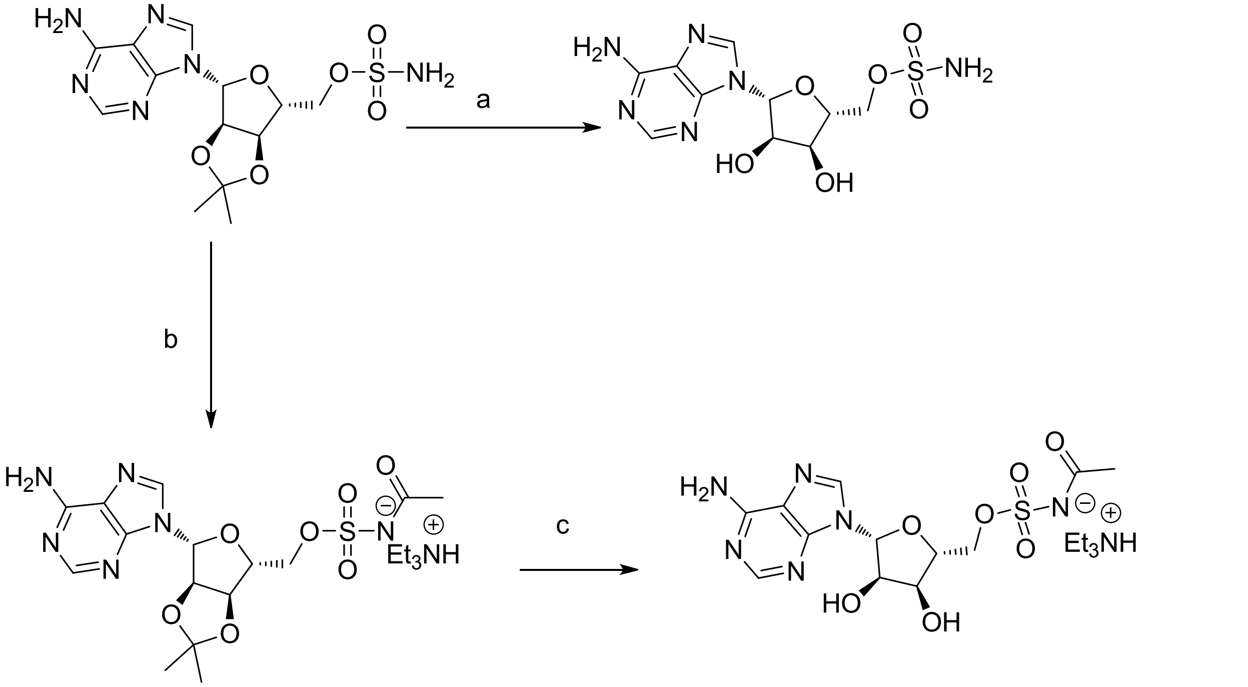

Reagent and reaction conditions: (a) TFA (80%) (b) N-acetoxysuccinimide, Cs_2CO_3 (2eq), DMF (d) TFA (80%).

5'-O-Sulfamoyladenosine (AMS)

To 5'-*O*-Sulfamoyl-2', 3'-*O*-isopropylideneadenosine (50 mg, 0.13 mmol) was added 80% aq TFA (2 mL). The resulting solution was stirred for 30 min at 0 °C then concentrated under reduced pressure. Purification by column chromatography on a Biotage Isolera using a gradient elution 15 to 20% MeOH in CH_2Cl_2 afforded the title compound as a white solid. Yield; 40mg, 89%, ^{1}H NMR (500 MHz, DMSO-*d6*) δ, 8.30 (s, 1H), 8.16 (s, 1H), 7.32 (s, 2H), 7.60 (s, 2H), 5.94 (d, J= 5.30 Hz,

1H), 5.44 (d, J= 5.20 Hz, 1H), 5.62 (d, J= 5.85 Hz, 1H), 4.63 (d, J=5.25 Hz, 1H), 4.30 (d, J=6.85 Hz, 1H), 4.23-4.21 (m, 2H), 4.17 (d, J=1.55 Hz), ^{13}C NMR (75 MHz, DMSO-*d6*), 156.5, 153.1, 149.9, 139.9, 119.6, 88.0, 82.0, 73.4, 70.8, 69.2.

5'-*O*-[N-acyl(sulfamoyl)]-2', 3'-*O*-isopropylideneadenosine triethylammonium salt.

To a solution of N-acetoxysuccinimide (0.86mmol) in DMF (10 mL) at 0 °C were added 5'-*O*-sulfamoyl-2',3'-*O*-isopropylideneadenosine[1] (1.29 mmol, 1.5equiv) and Cs_2CO_3 (1.72 mmol, 2.0 eq.). The reaction mixture was warmed to rt and stirred 16 h. The reaction was concentrated under reduced pressure and the crude material was chromatographed on a Biotage Isolera using EtOAc/MeOH/Et_3N (70:29:1) to afford the title compound as a white solid. Yield; 87%. ^{1}H NMR (300 MHz, DMSO-*d6*) δ 8.38 (s, 1H), 8.15 (s, 1H), 7.31 (s, 2H), 6.15 (d, J = 2.9 Hz, 1H), 5.35 (dd, J = 2.9, 6.1 Hz, 1H), 5.02 (d, J = 8.5 Hz, 1H), 4.37 (d, J = 2.5 Hz, 1H), 3.98 (d, J= 5.0Hz, 2H), 3.04 (q, J = 7.3 Hz, 6H), 1.72 (s, 3H), 1.54 (s, 3H), 1.32 (s, 3H), 1.16 (t, J = 7.3 Hz, 9H), ^{13}C NMR (75 MHz, CD_3OD), 179.2, 157.3, 155.9, 149.0, 140.1, 120.0, 113.9, 90.4, 85.2, 84.3, 80.4, 68.2,43.2, 26.1, 25.1, 23.7, 7.9.

5'-*O*-[N-acyl(sulfamoyl)] adenosine triethylammonium salt (Ac-AMS)

To 5'-*O*-[N-acyl(sulfamoyl)]-2', 3'-*O*-isopropylideneadenosine triethylammonium salt (0.50 g, 0.94mmol) was added 80% aq TFA (5 mL). The resulting solution was stirred for 30 min at 0 °C then concentrated under reduced pressure. Purification by column chromatography on a Biotage Isolera using EtOAc/MeOH/Et_3N (70:29:1) afforded the title compound as a pale yellow solid. Yield; 0.42 g, 89%. ^{1}H NMR (500 MHz, CD_3OD) δ 8.52 (s, 1H), 8.23 (s, 1H), 6.11 (d, J = 6.0 Hz, 1H), 4.69 (s, 1H), 4.42 (s, 1H), 4.34-4.31 (m, 3H), 3.20 (q, J = 7.0 Hz, 6H), 1.98 (s, 3H), 1.31 (t, J = 7.2 Hz, 9H), ^{1}H NMR (300 MHz, DMSO-*d6*) δ 8.40 (s, 1H), 8.14 (s, 1H), 7.27 (s, 2H), 5.91 (d, J = 6.0 Hz, 1H), 5.46 (s, 1H), 5.31 (s, 1H), 4.60 (s, 1H), 4.14 (s, 1H), 4.07-3.99 (m, 3H), 3.04 (q, J

= 7.0 Hz, 6H), 1.74 (s, 3H), 1.14 (t, J = 7.2 Hz, 9H), ^{13}C NMR (75 MHz, DMSO-*d6*), 175.5, 156.5, 153.1, 150.1, 139.8, 119.3, 93.6, 87.2, 83.2, 74.1, 71.4, 67.6, 46.2, 26.9, 9.5.

Crystallization conditions and procedures

Aspergillus fumigatus

Aspergillus fumigatus ACS1 in complex with adenosine-5'-propylphosphate (propyl AMP) was crystallized at 10 mg ml-1 in sparse matrix screen Morpheus (Molecular Dimensions) condition g9 (10% w/v PEG 20000, 20% v/v PEG MME 550, 0.02M carboxylic acids (sodium formate, ammonium acetate, trisodium citrate, sodium potassium L-tartrate, sodium oxamate, 0.1M bicine/Trizma base pH 8.5), with 1mM propyl AMP and 1mM TCEP (2). No additional cryo-protectant was used.

Candida albicans

Candida albicans ACS2 was crystallized in complex with propyl AMP at 10 mg/ml in sparse matrix screen MCSG1 (Microlytic/Anatrace) condition g10 (100mM Mg-formate, 15% (w/v) PEG 3350) with 1mM Propyl AMP and 1mM TCEP. 25% Ethylene glycol (v/v) was used as a cryoprotectant.

Coccidioides posadasii

Crystals of Coccidioides posadasii ACS1 in complex with propyl AMP were grown at 10 mg/ml ACS1 in sparse matrix screen Morpheus (Molecular Dimensions) condition a6 (10% w/v PEG 8000, 20% v/v ethylene glycol, 0.03M divalent cations (MgCl2, CaCl2), 0.1M MOPS/HEPES-Na pH7.5), with 1mM propyl-AMP and 1mM TCEP (2). No additional cryo-protectant was used.

Coccidioides immitis

Crystals of Coccidioides immitis ACS1 in complex with methyl AMP were grown at 10 mg/ml ACS1 in sparse matrix screen MCSG1 condition f11 (0.1M HEPES/NaOH, pH7.5, 0.2M Ammonium sulfate, 25% w/v PEG 3,350), with 1mM methyl AMP and 1mM TCEP. 15% (v/v) ethylene glycol was used as a cryoprotectant. Crystals containing methyl AMP and coenzyme A were grown in sparse matrix screen MCSG1 condition a6 (18.71% PEG 3,350, 0.2M Lithium acetate), with 1mM methyl AMP, 1mM TCEP, and 1mM coenzyme A. 20% (v/v) ethylene glycol was used as a cryoprotectant. Ethyl AMP containing crystals were grown in optimization conditions based on screen MCSG1 condition h3 (200mM lithium acetate, 18.32-19.1% PEG 3350), with 1mM ethyl-AMP and 1mM TCEP. Crystals containing a complex with ethyl AMP and coenzyme A were setup in similar conditions with the addition of 1mM coenzyme A. 20% (v/v) ethylene glycol was used as a cryoprotectant. Finally, crystals containing propyl AMP were grown in sparse matrix screen MCSG1 condition h5 (200 mM Potassium chloride, 20% (w/v) PEG 3350), with 1mM pro-pyl AMP and 1mM TCEP. 20% (v/v) ethylene glycol was used as a cryoprotectant.

Cryptococcus neoformans

Crystals of Cryptococcus neoformans ACS1 were generally grown at 10 mg/ml in optimization conditions based on screen Wizard Classic 1&2 (Rigaku Reagents) condition e8 (10-20% (w/v) PEG 8000, 0.1M Na/K phosphate, pH 5.8-7). 20% (v/v) ethylene glycol was used as a cryoprotectant. Where applicable, AMP esters were added at 1mM (propyl, ethyl, and butyl AMP), coen-zyme A at 1-2mM, acetyl AMS at 0.5mM, ATP/MgCl2 at 1mM and TCEP at 1mM. Crystals of apo/acetylated active site lysine 640 from Cryptococcus neoformans were grown in sparse matrix screen Morpheus (Rigaku Reagents) condition h3 (10.0% w/v PEG4,000, 20% glycerol, 0.02M amino acid mix, 0.1M MES/imidazole pH6.5) (34). No additional cryo-protectant was used.

Structure determination

The structures of the fungal ACS1 apo or with compound were collected between 2016-2020 at the Advanced Photon Source, beamlines 21-ID-F and G (CCD Rayonix MX-225 and MX-300 X-ray detector respectively), with the exception of 7KDS (Candida albicans ACS2) which was collected at the Canadian Lightsource (Pilatus3 S 6M X-ray detector). Data were reduced with XDS/XSCALE, (3) and solved by molecular replacement using Phaser with input models based upon previously solved crystal structures of fungal, yeast or bacterial ACS1. Iterative manual model building using Coot (4) and Phenix.Refine continued until R and Rfree converged (5). Model quality was validated using Coot and MolProbity (6). Structures were validated using Molprobity prior to deposition in the Protein Data Bank (See Crystallographic Table) (7, 8). Diffraction images are available on Integrated Resource for Reproducibility in Macromolecular Crystallography (http://www.proteindiffraction.org) (9-11).

References:

1. Qiao, C.; Gupte, A.; Boshoff, H. I.; Wilson, D. J.; Bennett, E. M.; Somu, R. V.; Barry, C. E., 3rd; Aldrich, C. C., 5'-O-[(N-acyl)sulfamoyl]adenosines as antitubercular agents that inhibit MbtA: an adenylation enzyme required for siderophore biosynthesis of the mycobactins. *J Med Chem* **2007,** *50* (24), 6080-94.
2. Miller KD, Pniewski K, Perry CE, Papp SB, Shaffer JD, Velasco-Silva JN, Casciano JC, Aramburu TM, Srikanth YVV, Cassel J, Skordalakes E, Kossenkov AV, Salvino JM, Schug ZT. (2021) Targeting ACSS2 with a transition-state mimetic inhibits triple-negative breast cancer growth. Cancer Res. 81, 1252-1264.
3. Gorrec F. The MORPHEUS protein crystallization screen. J Appl Crystallogr. 2009 Dec 1;42(Pt 6):1035-1042. doi: 10.1107/S0021889809042022. Epub 2009 Nov 7. PMID: 22477774; PMCID: PMC3246824.
4. Kabsch, W. (2010). Xds. Acta Crystallographica Section D: Biological Crystallography 66,125-132.
5. Emsley, P., Lohkamp, B., Scott, W.G. and Cowtan, K. (2010) Features and development of Coot. Acta Crystallographica Section D: Biological Crystallography 66, 486-501.
6. Adams, P.D., Afonine, P.V., Bunkóczi, G., Chen, V.B., Davis, I.W., Echols, N., Headd, J.J., Hung, L.W., Kapral, G.J., Grosse-Kunstleve, R.W. and McCoy, A.J. (2010) PHENIX: a com-prehensive Python-based system for macromolecular structure solution. Acta Crystallograph-ica Section D: Biological Crystallography 66, 213-221.
7. Chen, V.B., Arendall, W.B., Headd, J.J., Keedy, D.A., Immormino, R.M., Kapral, G.J., Murray, L.W., Richardson, J.S. and Richardson, D.C. (2010) MolProbity: all-atom structure

validation for macromolecular crystallography. Acta Crystallographica Section D: Biological Crystallog-raphy 66, 12-21.

8. Berman, H., Henrick, K. and Nakamura, H. (2003) Announcing the worldwide protein data bank. Nature Structural & Molecular Biology, 10, 980-980.

9. Berman, H.M., Westbrook, J., Feng, Z., Gilliland, G., Bhat, T.N., Weissig, H., Shindyalov, I.N. and Bourne, P.E. (2000) The protein data bank. Nucleic Acids Research 28, 235-242.

10. Grabowski M, Langner KM, Cymborowski M, Porebski PJ, Sroka P, Zheng H, Cooper DR, Zimmerman MD, Elsliger MA, Burley SK, Minor W (2016) A public database of macromolecu-lar diffraction experiments. Acta crystallographica. Section D, Structural biology 72 (Pt 11):1181-1193.

11. Grabowski M, Cymborowski M, Porebski PJ, Osinski T, Shabalin IG, Cooper DR, Minor W (2019) The Integrated Resource for Reproducibility in Macromolecular Crystallography: Expe-riences of the first four years. Struct Dyn 6: 064301.
